# Meiotic CENP-C supports centromere assembly and kinetochore recruitment in spermatogenesis

**DOI:** 10.1083/jcb.202512053

**Published:** 2026-06-30

**Authors:** Rachel S. Keegan, Dina Malkeyeva, Meg B. Weever, Elaine M. Dunleavy

**Affiliations:** 1 Centre for Chromosome Biology, University of Galway, Galway, Ireland

## Abstract

The centromere is the chromosomal site of kinetochore assembly, defined by the histone H3 variant CENP-A. In each cell cycle, the assembly and maintenance of CENP-A is functionally critical for chromosome segregation. In *Drosophila* male meiosis, CID (fly CENP-A) is assembled in two phases: prophase of meiosis I and postmeiosis II. Here, we investigate the dynamics of the assembly components CAL1 and CENP-C in prophase I and determine the requirements for the second assembly phase. In early prophase I, CENP-C functions with CAL1 to maintain the centromere. In late prophase I, CAL1 is undetectable at centromeres and CENP-C is not critical for centromere maintenance. Instead, CENP-C is crucial for meiotic kinetochore recruitment and function. This CENP-C pool also functions in CID assembly postmeiosis II, which is independent of CAL1. In addition to different functional pools of CENP-C, distinct pools of the CID protein persist in the male germline, and the synthesis of each pool is uncoupled from its cell cycle deposition timing.

## Teaser

In the fruit fly, the centromeric histone CENP-A is assembled in two phases: prophase of meiosis I and postmeiosis II. Keegan et al. show that a new pool of constitutive centromere protein CENP-C is loaded at the centromere in prophase of meiosis I. This pool is critical for kinetochore assembly and meiotic chromosome segregation.

## Introduction

Accurate segregation of replicated chromosomes is imperative for preserving genomic integrity and preventing aneuploidy. This is especially critical in the germline, where chromosome segregation during gametogenesis ensures the transmission of genetic information to future generations. Centromeres are unique chromosomal loci that are required for faithful chromosome segregation. They serve as platforms for kinetochore assembly, to which spindle microtubules attach during cell division (Kixmoeller et al., 2020; Fukagawa and Earnshaw, 2014). This locus is epigenetically specified by the incorporation of the histone H3 variant known as Centromere Protein A (CENP-A) (McKinley and Cheeseman, 2016; Allshire and Karpen, 2008). During DNA replication in the S phase, parental histones are diluted to half on each DNA strand and are replenished with newly synthesized histones at the replication fork. At centromeres, CENP-A is also diluted during DNA replication, but newly synthesized CENP-A does not load with the canonical histones during the S phase (Sundararajan and Straight, 2022). Instead, cells complete mitosis with only half the maximal occupancy of CENP-A nucleosomes, and new deposition occurs between the late telophase and early G1 phase (Jansen et al., 2007; Schuh et al., 2007).

*Drosophila melanogaster*, or the fruit fly, presents a simplified centromere composition, containing CENP-A (referred to as CID in flies), Chromosome Alignment Defect 1 (CAL1), and the inner kinetochore protein CENP-C (Kyriacou and Heun, 2023). In *Drosophila*, CENP-C is the only member of the multiprotein constitutive centromere-associated network complex existing in mammals (Foltz et al., 2006; Okada et al., 2006), and it bridges the centromere and outer kinetochore (Przewloka et al., 2011). CAL1 acts as the CID-specific chaperone and assembly factor (Chen et al., 2014), fulfilling the role of both Holliday Junction Recognition Protein (HJURP) and the Mis18 complex in mammals (Rowley and Jansen, 2025). In mitosis, CID, CAL1, and CENP-C are interdependent for their localization to the centromere (Erhardt et al., 2008). CID and CAL1 form a prenucleosomal complex that is recruited to the centromere by CENP-C. CAL1 recruits CENP-C, which in turn brings more prenucleosomal complexes of CID-CAL1, sustaining this epigenetic loop (Schittenhelm et al., 2010; Chen et al., 2014; Roure et al., 2019; Medina-Pritchard et al., 2020).

The mechanism of CENP-A deposition in meiosis, the specialized division in which homologous chromosomes separate to generate haploid gametes, is less well studied than in mitosis. The timing of cell cycle deposition is markedly different based on initial observations from diverse models. In female meiosis, CENP-A is assembled during, or prior to, prophase I in worms (*Caenorhabditis elegans*), starfish (*Patiria miniata*), mice (*Mus musculus*), and *Drosophila* (Monen et al., 2005; Swartz et al., 2019; Smoak et al., 2016; Tower et al., 2025; Fellmeth et al., 2023). In the *Drosophila* male germline, new CID assembly occurs at two distinct windows: initially, during meiotic prophase I; and later, immediately after meiosis II (Dunleavy et al., 2012; Raychaudhuri et al., 2012). A recent study indicates that CENP-A assembly in prophase I is conserved in mammalian spermatogenesis (Štiavnická et al., 2025). In *Drosophila* males, both CAL1 and CENP-C are required for the first meiotic loading event, yet both proteins show unexpected localization dynamics at centromeres (Dunleavy et al., 2012; Raychaudhuri et al., 2012). CAL1 is progressively reduced during prophase I and is undetectable for the remainder of meiosis. Contrastingly, centromeric CENP-C increases in prophase I and is undetectable in postmeiosis II spermatids, coincident with the second phase of CID assembly. Remarkably, CID is one of the few histones retained in the mature sperm of *Drosophila*, surviving histone-to-protamine exchange during spermiogenesis (Dunleavy et al., 2012; Raychaudhuri et al., 2012; Dubruille et al., 2025). Here, we investigate the significance of the unusual CAL1 and CENP-C dynamics during the first assembly phase in prophase I and determine the previously unexplored requirements for the second assembly phase on spermatids.

## Results

### Haploid spermatids have CID levels comparable to diploid spermatogonia

**Figure 1.**
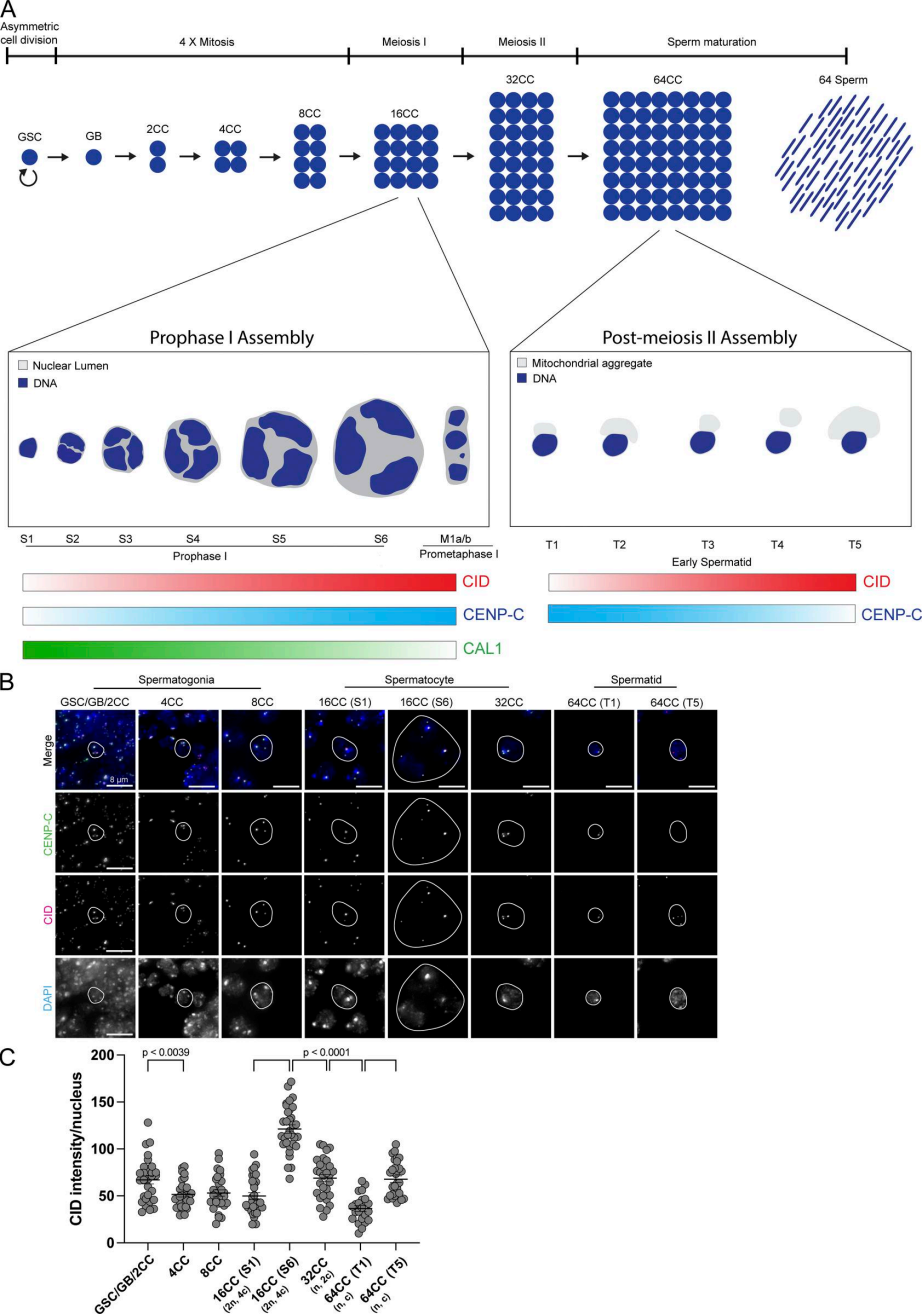
***Drosophila melanogaster* spermatogenesis and centromere assembly dynamics during meiosis. (A)** GSCs divide asymmetrically generating a self-renewing GSC and a differentiating daughter cell called a GB. Four mitotic divisions with incomplete cytokinesis generate a CC of 16 primary spermatocytes. Prophase I of meiosis is subdivided into six stages (S1 to S6), characterized by a massive expansion in nuclear volume, decondensation of DNA, and the appearance of three chromosome territories (Cenci et al., 1994). CID and CENP-C are assembled onto the centromere during prophase I, coinciding with a loss of chaperone CAL1. Prometaphase I nuclei (stage M1a/b) are arranged as 3–4 condensed chromosome territories. Meiotic cells divide twice to separate homologous chromosomes and sister chromatids in meiosis I and II, respectively. CID is assembled again after meiosis II in stages T1 to T5 of the 64CC early spermatids, during which mitochondrial material is also removed from the cells. CENP-C is progressively lost with new CID deposition and is undetectable by the stage T5. The resulting bundle of 64 early spermatids undergoes drastic chromatin remodeling and individualizes to form needle-shaped mature sperm nuclei. **(B)***Drosophila* wild-type pupal testes stained with anti-CENP-C (green), anti-CID (magenta), and DAPI (blue). Representative images of cell stages during spermatogenesis from the 2CC spermatogonia to T5 stage early spermatids (64CC). A representative nucleus has been outlined in white for each stage. Cells were staged based on nuclear appearance and relative distance from the testis apex. S1 and S6 show early and late prophase I, respectively. Scale bars = 8 µm. **(C)** Quantitation showing the relative fluorescent intensity per nucleus of the CID during each stage of spermatogenesis, with respective P values annotated on the graph. Data were tested for normality using a Shapiro–Wilk test and analyzed using parametric Welch’s *t* tests accordingly. Ploidy (2n, n) and DNA content at specific stages are indicated. *n* = 30–40, where *n* represents the number of cells analyzed from at least three independent replicates. Error bars = SEM. GB, gonialblast; CC, cell cyst.

**Figure S1.**
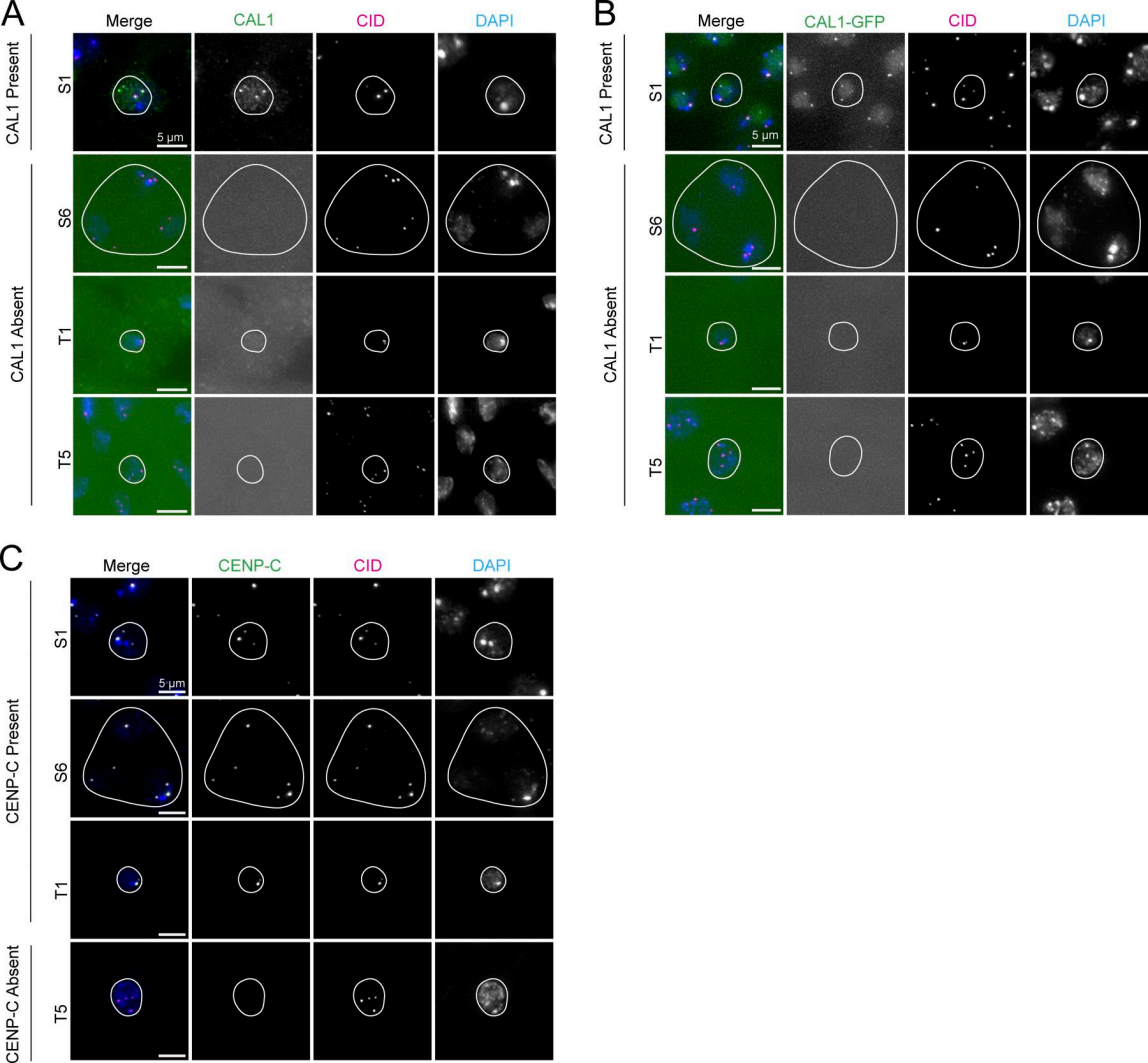
**CAL1 and CENP-C recruitment dynamics during meiosis and in the early spermatids. (A)** Pupal testes stained with anti-CAL1 (green) and anti-CID (magenta) antibodies, and DAPI (blue). CAL1 is present at the centromeres of S1 stage cells (16CC) and absent from the centromeres of S6 (16CC), T1, and T5 (64CC). CAL1 is also visible in the nucleolus of S1 cells. Scale bars = 5 µm. **(B)** Pupal testes from CAL1-GFP flies stained with anti-CID antibody (magenta) and DAPI (blue), showing the presence at the S1 stage and in the nucleolus, as well as an absence from later stages (S6, T1, and T5). Scale bars = 5 µm. **(C)** Pupal testes stained with anti-CENP-C (green) and anti-CID (magenta) antibodies, and DAPI (blue). CENP-C is present at the centromeres of S1, S6, and T1 stage cells, but absent from T5 stage centromeres. Scale bars = 5 µm.

*Drosophila* testes provide a spatiotemporal array encompassing all stages of spermatogenesis, with germline stem cells (GSCs) located in the apex and later developing stages toward the distal end (Fig. 1 A). *Drosophila* males undergo achiasmatic meiosis (Adams et al., 2020), lacking chromosomal features such as synapsis, homologous recombination, and chiasmata formation (McKee et al., 1992). To account for these differences, an alternative nomenclature has been introduced to distinguish stages of prophase I (Cenci et al., 1994). This convention subdivides prophase I into stages S1 to S6, characterized by an expansion in nuclear volume and the appearance of distinct chromosome territories into which homologous chromosome pairs are localized. Immediately after meiosis II, spermatids are described in stages T1 to T5, based on the appearance of a mitochondrial aggregate. As previously described (Dunleavy et al., 2012; Raychaudhuri et al., 2012), centromeric CAL1 progressively disappears during meiotic prophase I (Fig. S1, A and B), between S1 and S4, corresponding to a gradual increase in CID and CENP-C at centromeres (Fig. 1 A). After meiosis II, CID gradually increases during T1–T5 stages in spermatids, yet CENP-C progressively disappears and is absent at T5 (Figs. 1 A and S1 C).

To assess overall CID dynamics across the germline, centromeric CID intensity was measured in spermatogonial 2-cell cysts (2CC, including GSC and gonialblast pairs), 4-cell cysts (4CC), 8-cell cysts (8CC), spermatocytes at early prophase I (16CC at S1) and at late prophase I (16CC at S6), 32-cell cysts that have completed meiosis I (32CC), and 64-cell cysts that have completed meiosis II (64CC at T1 and T5) (Fig. 1, A–C). A decrease in CID was measured between 2CC and 4CC, consistent with previous reports that GSCs harbor ∼1.5-fold more CID than daughter cells (Ranjan et al., 2019; Kochendoerfer et al., 2023; Tower et al., 2025). CID levels were then quantitatively maintained throughout the subsequent mitotic divisions in 4CC, 8CC, and upon entry into meiosis in 16CC at the S1 stage. Between S1 and S6 stages of prophase I, an ∼2.5-fold increase in CID was noted, in line with the first phase of CID assembly occurring at this stage (Dunleavy et al., 2012; Raychaudhuri et al., 2012). After the first meiotic division, CID levels were reduced by ∼50% in 32CC. This is expected given homologous chromosome segregation in meiosis I and a reduction in ploidy. A further proportional reduction was measured in 64CC at the T1 spermatid stage, congruous with sister chromatid segregation. The T1 stage of spermatid development marked the lowest levels of CID measured throughout spermatogenesis. Consistent with previous findings of a second phase of CID assembly in postmeiotic spermatids (Dunleavy et al., 2012), an increase in CID intensity (1.8-fold) was observed between T1 and T5 stages. Overall, CID levels in haploid T5 spermatids were equivalent to the levels in diploid 2CC stage spermatogonia. This result indicates that despite two rounds of chromosome segregation in meiosis, the CID level in haploid spermatids is comparable to diploid premeiotic cells due to two unique phases of CID assembly occurring during spermatogenesis, with the first providing significantly higher-than-expected quantities (2.5-fold measured increases compared with the expected twofold).

### CENP-C localization in meiosis is uncoupled from CID and CAL1 levels

**Figure S2.**
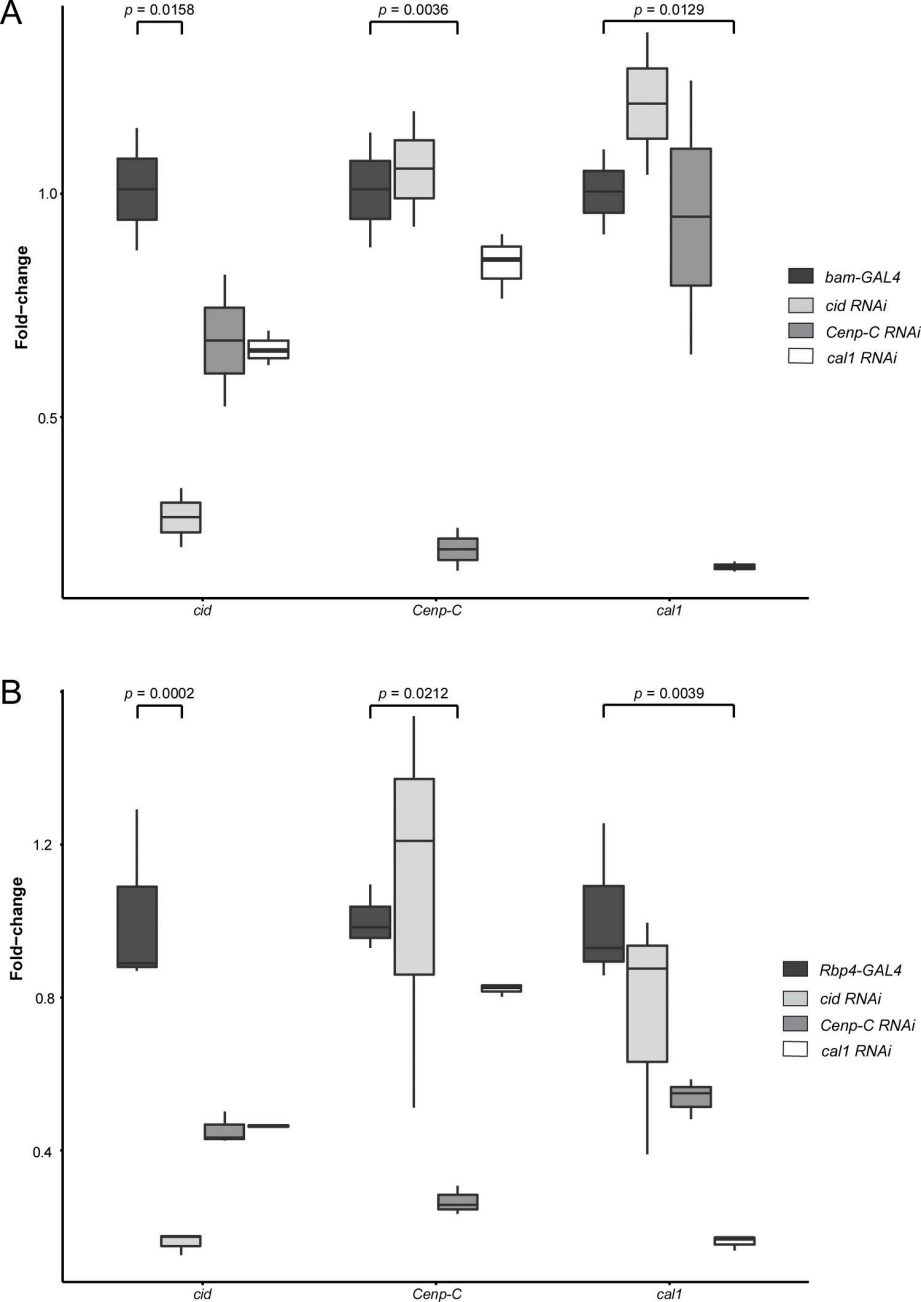
**Efficiency of *cid*, *Cenp-C*, and *cal1* knockdown in the testes of P4−P5 pupae via quantitative PCR. (A and B)** Results of qPCR showing the relative fold change between the target and the reference (*αTub84B* and *eEF1α2*) mRNA levels in the testes of the control ([A] *bam-GAL4* or [B] *Rbp4-GAL4*) and the *cid*, *Cenp-C*, or *cal1* knockdown pupae induced by GAL4 driver under *bam* or *Rbp4* promoter. Horizontal lines within the boxes denote median values; boxes indicate the interquartile range; and whiskers represent the highest and lowest values. *cid* was downregulated 3.7-fold in the *bam-GAL4; cid RNAi* testes (P = 0.016, *n* = 2) and 6.4-fold in the *Rbp4-GAL4; cid RNAi* testes (P = 0.0002, *n* = 3). *Cenp-C* was reduced 4.9-fold in the *bam-GAL4; Cenp-C RNAi* samples (P = 0.0036, *n* = 2) and 3.8-fold in the *Rbp4-GAL4; Cenp-C RNAi* (P = 0.0212, *n* = 3). *cal1* was downregulated approximately sixfold in both sets of *cal1* RNAi experiments (P = 0.0129 in the *bam-GAL4* experiment and P = 0.0039 in the *Rbp4-GAL4* experiment; *n* = 3 in both experiments).

**Figure 2.**
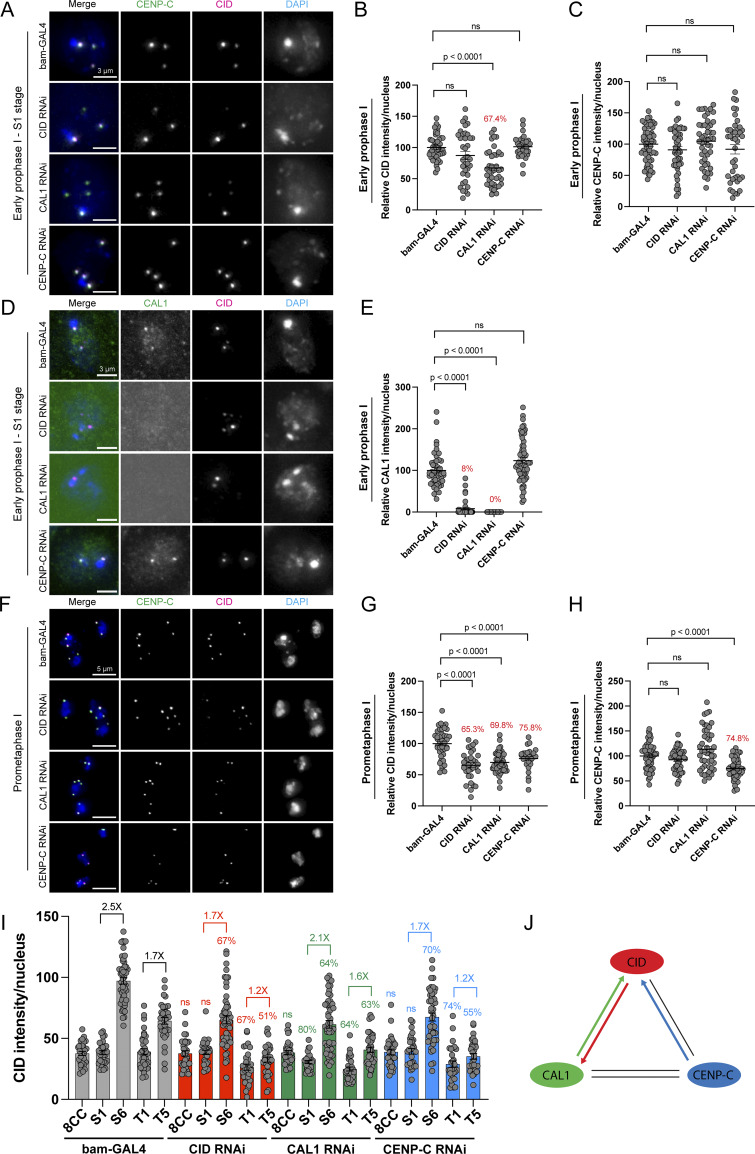
**Requirements of CID, CAL1, and CENP-C for their recruitment and stability in meiosis I. (A)** Pupal testes stained with anti-CENP-C (green) and anti-CID (magenta) antibodies, and DAPI (blue). Representative images of early prophase I (S1 stage) cells from each of the genetic lines (bam-GAL4, CID RNAi, CAL1 RNAi, and CENP-C RNAi). Scale bars = 3 µm. **(B)** Quantitation showing the relative fluorescent intensity per nucleus of CID at S1 in early prophase I. *n* = 46, 37, 39, 34. Here, and throughout this figure, *n* represents the number of cells analyzed from at least three independent replicates; data were checked for normality using the Shapiro–Wilk tests and analyzed using either Welch’s or Mann–Whitney tests. Error bars = SEM. **(C)** Quantitation showing the relative fluorescent intensity per nucleus of CENP-C at S1 in early prophase I. *n* = 57, 47, 30, 31. Error bars = SEM. **(D)** Early prophase I (S1 stage) cells stained with anti-CAL1 (green) and anti-CID (magenta) antibodies, and DAPI (blue). Scale bars = 3 µm. **(E)** Quantitation showing the relative fluorescent intensity per nucleus of the histone chaperone CAL1 at S1 in early prophase I. *n* = 46, 37, 55, 61. Error bars = SEM. **(F)** Prometaphase I cells stained with anti-CENP-C (green) and anti-CID (magenta) antibodies, and DAPI (blue). Each panel shows one nucleus, visible as three to four chromosome territories at prometaphase I. Scale bars = 5 µm. **(G)** Quantitation showing the relative fluorescent intensity per nucleus of CID at prometaphase I. *n* = 42, 33, 45, 27. Error bars = SEM. **(H)** Quantitation showing the relative fluorescent intensity per nucleus of CENP-C at prometaphase I. *n* = 50, 50, 46, 49. Error bars = SEM. **(I)** Quantitation showing the fluorescent intensity of CID per nucleus from 8CC, early prophase I (S1), late prophase I (S6), and the spermatid stages T1 and T5 for bam-GAL4 (shown in gray), CID RNAi (red), CAL1 RNAi (green), and CENP-C RNAi (blue). Fold changes in the first phase of CID assembly are annotated between S1 and S6 for each line. Similarly, fold changes are shown between T1 and T5 for the second phase of assembly. Significant percent reductions in CID intensities relative to the same cell stage from the bam-GAL4 control are indicated for each RNAi line. *n* = 30–50. Error bars = SEM. **(J)** Model depicting the interdependencies for CID, CAL1, and CENP-C recruitment during meiosis I. Arrows represent a complete dependency between these proteins for their centromeric recruitment, with the color indicating the direction of this dependency. Black lines indicate incomplete dependency.

In mitosis, CID, CAL1, and CENP-C are interdependent for centromere localization (Erhardt et al., 2008; Roure et al., 2019). To examine interdependencies in meiosis, the GAL4-UAS system was used to target *cid*, *cal1*, or *Cenp-C* mRNA for degradation, thereby reducing the pool of proteins available for deposition at centromeres. RNAi was induced using the germline-specific driver *bam-GAL4* (active in 8CC spermatogonia and 16CC spermatocytes), and the knockdowns were confirmed by quantitative PCR (Fig. S2 A). Centromeres of pupal testes were stained with anti-CID, anti-CENP-C, or anti-CAL1 antibodies. CID intensity was first quantified at the S1 stage, representing the start of CID assembly (Fig. 1 C). Quantitation of total centromeric CID intensity per nucleus showed normal levels in CID and CENP-C RNAi, confirming that CID assembly is not yet impacted (Fig. 2, A and B). CID and CENP-C RNAi displayed normal CENP-C levels, corresponding to normal levels of CID (Fig. 2, A and C). Contrastingly, centromeric CID was significantly reduced in CAL1 RNAi, indicating that CAL1 is required to stabilize CID already incorporated at centromeres at the S1 stage (Fig. 2 A and B). Despite this loss of CID, CENP-C levels were quantified as normal at S1 in the CAL1 RNAi (Fig. 2, A and C). This indicates that CID is not required to proportionally maintain the centromeric association of CENP-C in early prophase I. In the CAL1 RNAi, CAL1 was completely depleted from centromeres at S1 (Fig. 2, A and B), likely reflecting its higher turnover rate and lack of stable integration at the centromere (Mellone et al., 2011; Roure et al., 2019). Surprisingly, CAL1 was almost undetectable at the centromere in CID RNAi, demonstrating that CAL1 requires newly synthesized CID for its localization to centromeres at S1, where it functions to stabilize nucleosomal CID (Fig. 2, D and E). Contrastingly, in the CENP-C RNAi, CAL1 localized to centromeres and in the nucleolus as expected (Kwenda et al., 2016) (Fig. 2, D and E). In summary, at the S1 stage, CAL1 is required for the stability of nucleosomal CID, and CENP-C is maintained at centromeres in a manner that is not strictly dependent on CID and CAL1 levels.

We next examined CID intensity at prometaphase I, immediately after S6 and representing the maximal level of CID assembly (Fig. 1 C). Primary spermatocytes at prometaphase I were identified based on nuclear morphology, typically comprising 3–4 condensed chromosome territories (Fig. 2 F). Quantitation of total centromeric CID intensity per nucleus measured an ∼35, 30, and 25% reduction in CID, CAL1, and CENP-C RNAi lines, respectively (Fig. 2 G), consistent with previous studies (Dunleavy et al., 2012). A 2.5-fold increase in total CID normally occurs in prophase I (Fig. 1 C and Fig. 2 I). CID, CAL1, and CENP-C RNAi display 1.7-, 2.1-, and 1.7-fold changes in CID, respectively, indicating that new CID assembly is affected in all three lines (Fig. 2 I). Next, centromeric CENP-C localization was quantified per nucleus in CID, CAL1, and CENP-C RNAi to establish whether inner kinetochore recruitment was impacted. CENP-C was reduced by ∼30% in the CENP-C RNAi, consistent with the 25% reduction in CID (Fig. 2, F–H). However, the CENP-C level was unchanged in CID and CAL1 RNAi, despite previously observed reductions of 35 and 30% in centromeric CID (Fig. 2, F–H). This result indicates that despite a reduction in the total amount of CID at centromeres in all three RNAi lines, CENP-C assembly was affected only when it was targeted directly in the CENP-C RNAi. Furthermore, CID that is stably incorporated from prior mitotic divisions is sufficient to permit normal CENP-C recruitment in meiosis. Taken together, analyses of both early (S1) and late (prometaphase I) stages demonstrate that CENP-C localization in meiosis is not strictly dependent on CAL1, and can occur normally even with reduced CID. Thus, unlike mitosis, the interdependency feedback loop of centromere localization between CID, CAL1, and CENP-C is incomplete (Fig. 2 J).

### CENP-C is more important for meiotic kinetochore recruitment and microtubule attachment than the underlying CID level

**Figure 3.**
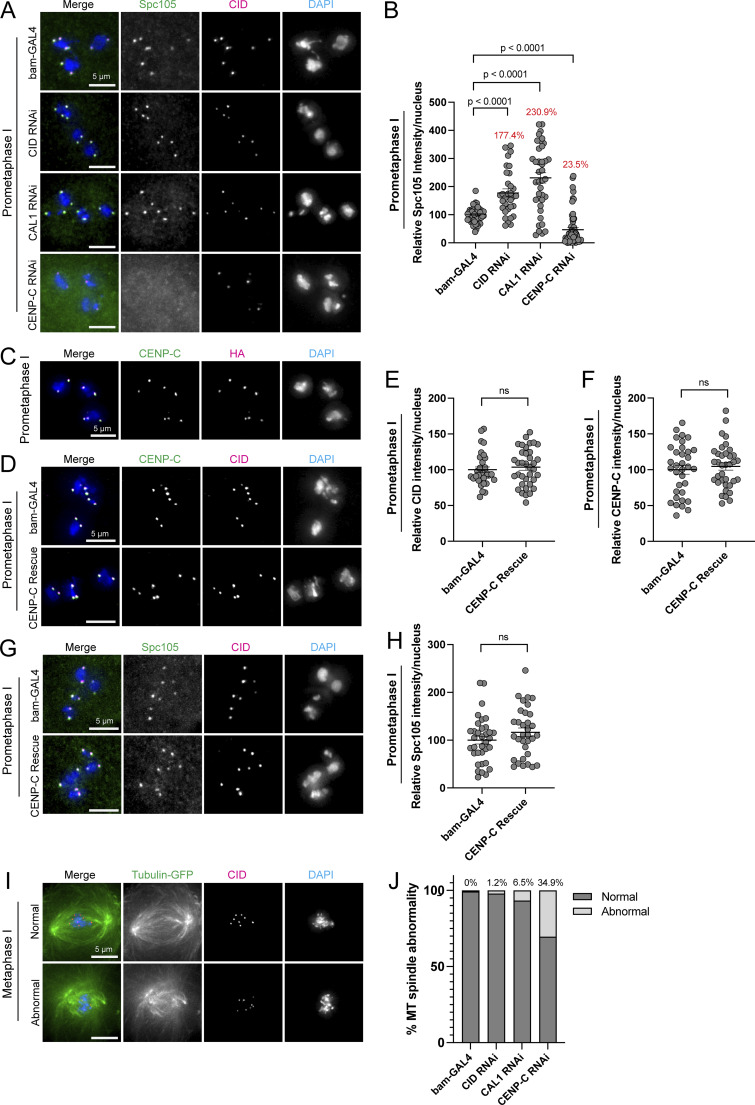
**Outer kinetochore recruitment and microtubule spindle assembly in CID, CAL1, and CENP-C RNAi. (A)** Pupal testes stained with anti-Spc105 (green) and anti-CID (magenta) antibodies, and DAPI (blue). Representative images of prometaphase I cells from each of the genetic lines (bam-GAL4, CID RNAi, CAL1 RNAi, and CENP-C RNAi). Scale bars = 5 µm. **(B)** Quantitation showing the relative fluorescent intensity per nucleus of the outer kinetochore component Spc105 (homolog of KNL1 in humans) at prometaphase I. *n* = 52, 32, 40, 57. Here, and throughout this figure, *n* represents the number of cells analyzed from at least three independent replicates; data were checked for normality using Shapiro–Wilk tests and analyzed using either Welch’s or Mann–Whitney tests. Median intensity is shown for CENP-C RNAi (23.5%). Error bars = SEM. **(C)** Prometaphase I cell from CENP-C rescue flies stained with anti-CENP-C (green), anti-HA (magenta), and DAPI. Scale bar = 5 µm. **(D)** Representative images of prometaphase I cells from bam-GAL4 and CENP-C rescue testes showing anti-CENP-C (green), anti-CID (magenta), and DAPI (blue). Scale bar = 5 µm. **(E)** Quantitation showing relative fluorescent intensity per nucleus of CID in bam-GAL4 control and CENP-C rescue cells at prometaphase I. *n* = 34, 36. **(F)** Quantitation showing relative fluorescent intensity per nucleus of CENP-C. *n* = 34, 36. **(G)** Representative images of prometaphase I cells from bam-GAL4 and CENP-C rescue testes showing anti-Spc105 (green), anti-CID (magenta), and DAPI (blue). Scale bar = 5 µm. **(H)** Quantitation showing relative fluorescent intensity per nucleus of Spc105. *n* = 35, 33. **(I)** Representative images of normal and abnormal microtubule spindles showing tubulin-GFP (green), anti-CID (magenta), and DAPI (blue). Scale bar = 5 µm. **(J)** Quantitation of microtubule spindle abnormality in each knockdown line with meiosis I and II pooled together. KNL1, Kinetochore Scaffold 1.

To determine the functional importance of CID assembly in prophase I, recruitment of the outer kinetochore protein Spindle Pole Component 105 (Spc105) was monitored in CID, CAL1, and CENP-C RNAi (Fig. 3. A and B). Spc105 is homologous to human Kinetochore Scaffold 1 and is required to maintain chromosome orientation and cohesion, recruit NDC80, and establish microtubule attachments (Joshi et al., 2024). Strikingly, the Spc105 signal at centromeres in prometaphase I was greatly reduced only in the CENP-C RNAi, indicating that the assembly of new CENP-C in prophase I is necessary to recruit the outer kinetochore (Fig. 3 A and B). Thus, CENP-C inherited from prior divisions is not sufficient to establish intact meiotic kinetochores. Importantly, Spc105 recruitment was restored in a CENP-C rescue experiment in which CENP-C RNAi was performed in a line expressing RNAi-resistant HA-tagged CENP-C under *UAS* control (Fig. 3, C and H). HA-CENP-C was confirmed to be expressed and incorporated at prometaphase I centromeres using anti-HA staining (Fig. 3 C). CID and CENP-C levels were also confirmed to be normal in the rescue flies, demonstrating that CENP-C deficiency drives the loss of Spc105 recruitment (Fig. 3, D–F). In contrast to the CENP-C RNAi, Spc105 was present at levels unexpectedly higher than the control in CID and CAL1 RNAi (Fig. 3, A and B). This result suggests that despite reduced CID at centromeres, CENP-C recruitment occurs in a manner that stimulates further Spc105 recruitment.

Assembly of bipolar meiotic spindles was next monitored in the CID, CAL1, and CENP-C RNAi lines expressing GFP-tagged tubulin and immunostained for CID (Fig. 3, I and J). Spindles lacking a defined bipolar structure and without direct contact to centromeres were scored as abnormal for both meiotic divisions. Quantitation of normal and abnormal meiotic spindles revealed that 34.9% of meiotic figures were abnormal in the CENP-C RNAi, compared with 1.2 and 6.5% in the CID and CAL1 RNAi, respectively. Though CID and CAL1 RNAi cells have reduced centromeric CID, an apparent compensatory recruitment of CENP-C (and Spc105) prevented spindle abnormalities. Taken together, the assembly of meiotic CENP-C appears to dictate the level of kinetochore recruitment, independent of the underlying CID level. Additionally, a deficiency in CID or CAL1 can be tolerated, providing that sufficient CENP-C is recruited.

### CENP-C is crucial for accurate meiotic chromosome segregation

**Figure 4.**
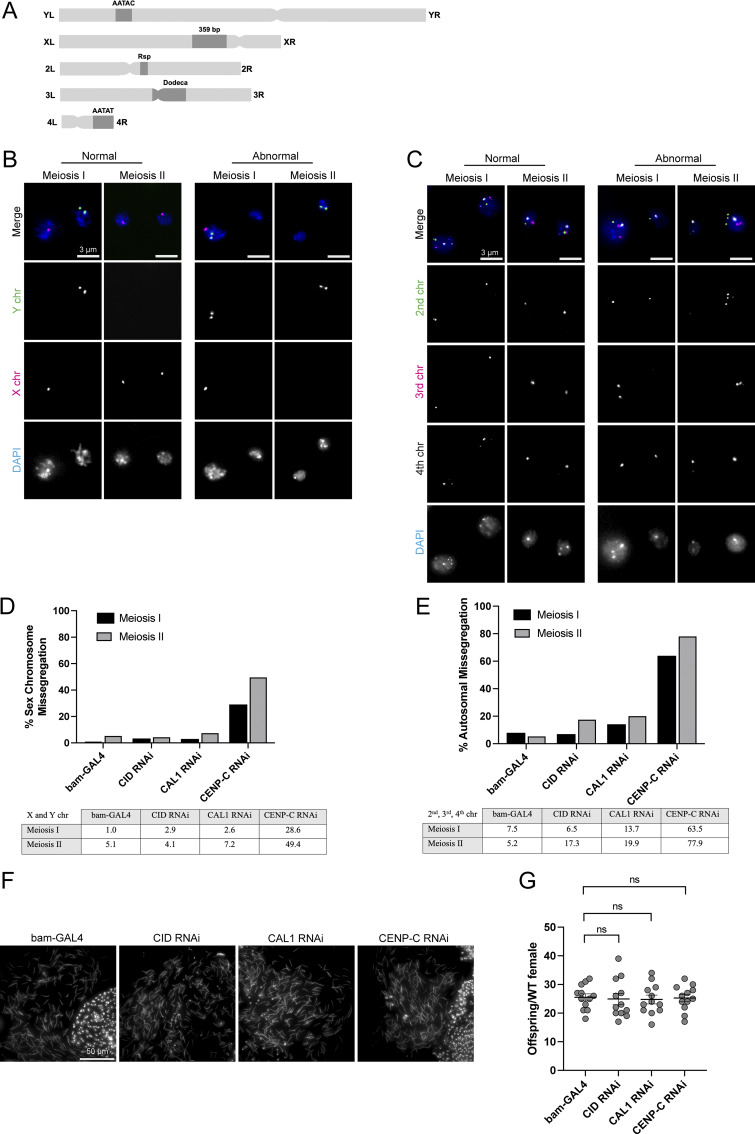
**Meiotic chromosome missegregation and overall fertility in CID, CAL1, and CENP-C RNAi. (A)** Schematic of the fluorescence *in situ* hybridization probes used to follow chromosome segregation through the meiotic divisions. Probes are specific for each of the three autosomes and the sex chromosome pair (X and Y) in *Drosophila melanogaster*. **(B)** Fluorescence *in situ* hybridization with Y chromosome (green) and X chromosome (magenta) probes, performed on pupal testes from the control and three knockdown lines (bam-GAL4, CID RNAi, CAL1 RNAi, and CENP-C RNAi). Representative images of the normal and abnormal chromosome segregation patterns using the X and Y probes in meiosis I and II. Scale bar = 3 µm. **(C)** Fluorescence *in situ* hybridization with second (Rsp probe), third (Dodeca satellite), and fourth (AATAT repeat) autosomes, showing normal and abnormal segregation patterns in meiosis I and II. Scale bar = 3 µm. **(D)** Quantitation displaying the percentage of sex chromosome missegregation for the control and each of the knockdown lines (bam-GAL4, CID RNAi, CAL1 RNAi, and CENP-C RNAi) after meiosis I and II. *n* = 4,470. Here, and throughout this figure, *n* represents the number of cells analyzed from at least three independent replicates. **(E)** Quantitation of the percentage of autosomal missegregation for each genetic line after meiosis I and II. *n* = 3,830. **(F)** Representative images displaying mature sperm nuclei stained with DAPI (blue) that have spilt out of the seminal vesicles of 3- to 5-day-old males from each genetic line (bam-GAL4, CID RNAi, CAL1 RNAi, and CENP-C RNAi). *n* = 47, 39, 43, 44, respectively, for each fly line. Scale bar = 50 µm. **(G)** Results of fertility assays in which males of each genetic line have been crossed to wild-type (Oregon R) females. The offspring per female was counted from three independent replicates. Data were checked for normality using Shapiro–Wilk tests and analyzed using either Welch’s or Mann–Whitney tests, accordingly. Rsp, Responder.

**Figure S3.**
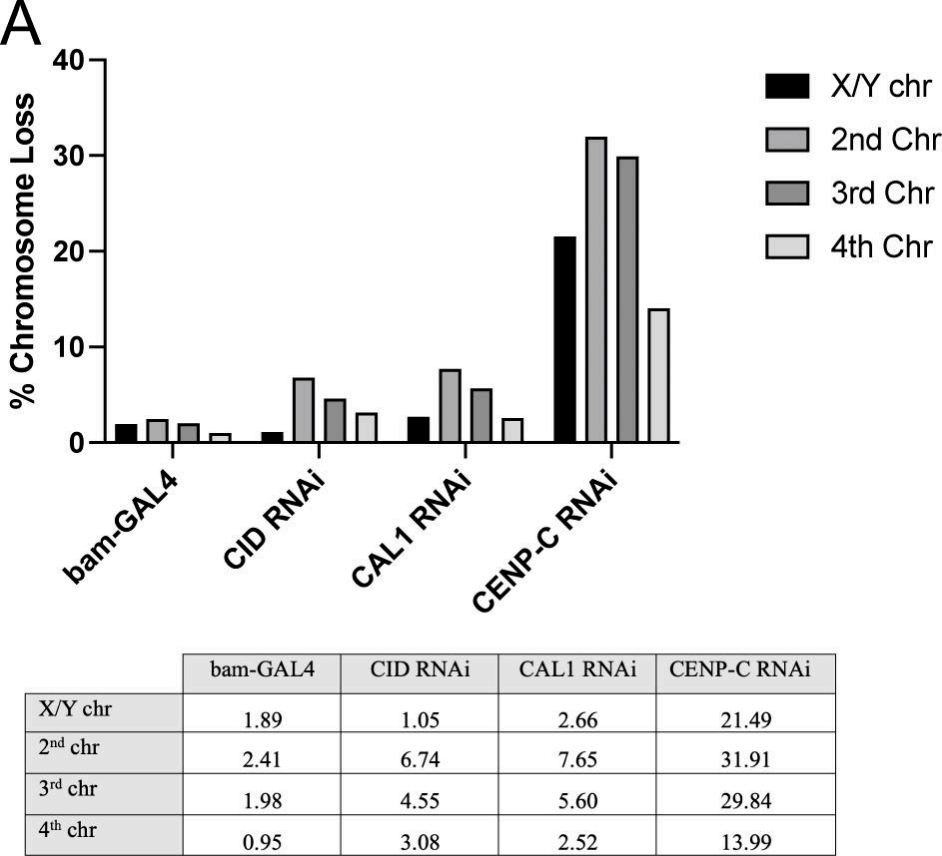
**Chromosome loss upon CID, CAL1, and CENP-C RNAi.** **(A)** Quantitation of the percentage of chromosome loss for each homologous pair (X/Y, second, third, and fourth chromosomes) in each knockdown line (bam-GAL4, CID RNAi, CAL1 RNAi, and CENP-C RNAi). *n* = 8,300, where *n* represents the number of cells analyzed from at least three independent replicates.

To investigate the consequences of centromere deficiencies on meiotic chromosome segregation, missegregation rates were examined in CID, CAL1, and CENP-C RNAi lines. To track segregation of homologous sex chromosomes in meiosis I, FISH probes recognizing sequences specific to the X (359-bp repeat) and Y (AATAC repeat) chromosomes were used (Fig. 4, A and B). In primary spermatocytes, the Y chromosome probe typically displays two foci, whereas the X chromosome is visualized as a single focus, consistent with Tsai et al. (2011). Pairs of secondary spermatocytes that just completed homologous sex chromosome segregation in meiosis I were scored as normal if one nucleus had an X chromosome and the other a Y chromosome. Any variation to this pattern was scored as abnormal. Pairs of spermatids that just completed meiosis II were scored as normal if both nuclei had an X chromosome, or both nuclei had a Y chromosome. Again, any variation to this pattern was scored as abnormal for meiosis II. To track segregation of autosomes in meiosis I and II, we used FISH probes specifically recognizing sequences on the second (Responder), third (Dodeca), or fourth (AATAT repeat) chromosomes (Fig. 4, A and C). Pairs of spermatocytes that completed meiosis I or II were scored as normal if each nucleus had one of each focus, representing the segregation of homologous chromosomes and sister chromatids, respectively. Any variation to this pattern in meiosis I or II was scored as abnormal. For the sex chromosomes (Fig. 4 D), the highest chromosome missegregation rate was measured for CENP-C RNAi, in both meiosis I (28.6%) and II (49.4%). For the autosomes (Fig. 4 E), the highest chromosome missegregation rate was again measured for CENP-C RNAi, in both meiosis I (63.5%) and II (77.9%). Overall, CENP-C RNAi showed a chromosome loss rate of ∼90%, with just 10% of postmeiotic spermatids displaying a normal karyotype (Fig. S3). Elevated rates of autosome missegregation were counted in the CID RNAi (6.5% for meiosis I and 17.3% for meiosis II) and were increased in the CAL1 RNAi (13.7% for meiosis I and 19.9% for meiosis II). Thus, CID and CAL1 RNAi displayed missegregation rates higher than the control, but consistently lower than CENP-C RNAi. These results demonstrate that CENP-C levels in meiosis are crucial for completing correct chromosome segregation. Finally, the impact of CID, CAL1, and CENP-C depletion during meiosis on male fertility was tested. Despite observed deficiencies in centromeric CID, individualized mature sperm were visible at levels comparable to the control in the seminal vesicles for all three RNAi lines (Fig. 4 F). Fertility tests did not measure any defect in the number of adult progenies produced compared with the bam-GAL4 control (Fig. 4 G). In summary, despite reduced meiotic CID levels and high rates of meiotic chromosome missegregation, particularly in the CENP-C RNAi, CID, CAL1, and CENP-C depletion did not impact male fertility.

### The second postmeiotic phase of CID assembly cannot recover deficient assembly in the first phase

**Figure 5.**
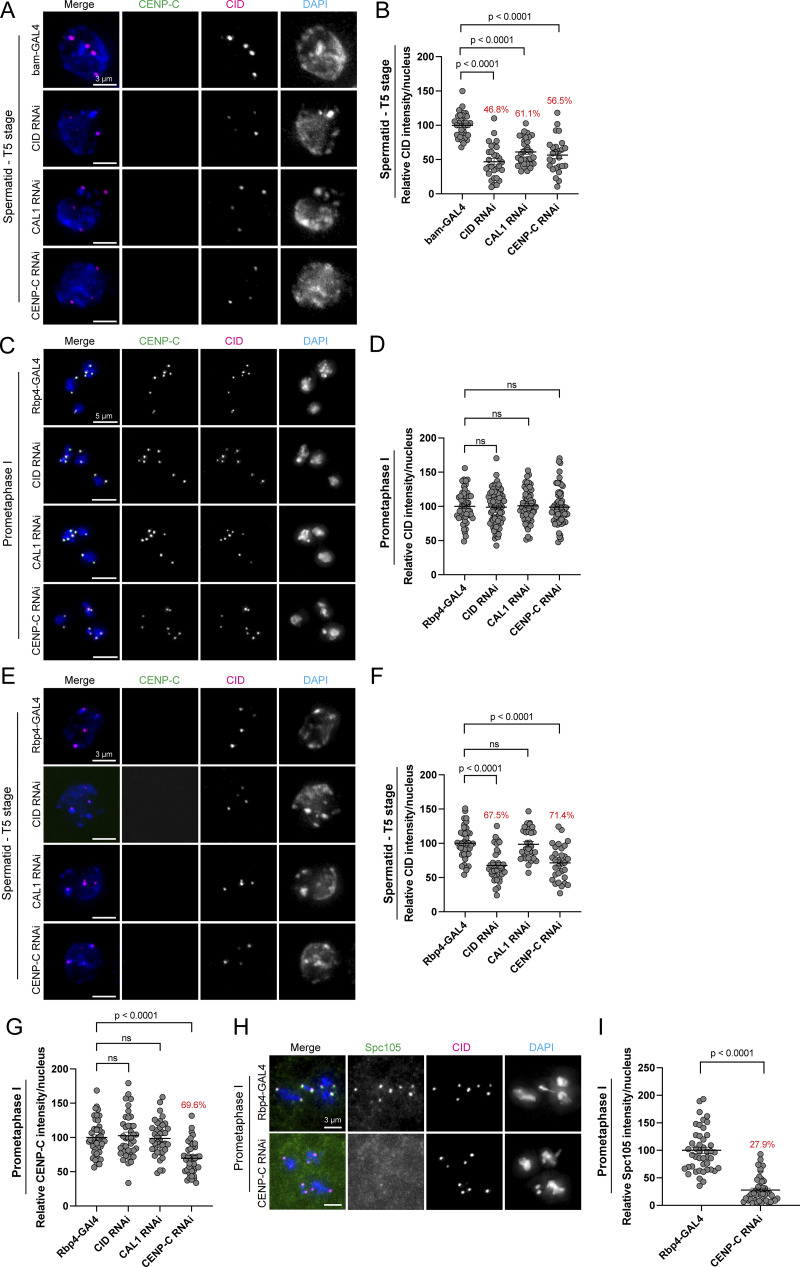
**Requirements for the second phase of CID assembly after meiosis II. (A)** Pupal testes stained with anti-CENP-C (green) and anti-CID (magenta) antibodies, and DAPI (blue). Representative images of T5 spermatid nuclei from each of the genetic lines (bam-GAL4, CID RNAi, CAL1 RNAi, and CENP-C RNAi). CENP-C staining is notably absent from each cell, verifying T5 stage spermatid identity. Scale bars = 3 µm. **(B)** Quantitation showing the relative fluorescent intensity per nucleus of CID. *n* = 32, 29, 27, 26. Here, and throughout this figure, *n* represents the number of cells analyzed from at least three independent replicates; data were checked for normality using Shapiro–Wilk tests and analyzed using either Welch’s or Mann–Whitney tests. Error bars = SEM. **(C)** Pupal testes from a new GAL4 driver, *Rbp4-GAL4* (expressed between S1 and S6), crossed to each RNAi line. Representative images of prometaphase I cells stained with anti-CENP-C (green) and anti-CID (magenta) antibodies, and DAPI (blue) from each of the genetic lines (Rbp4-GAL4, CID RNAi, CAL1 RNAi, and CENP-C RNAi). Scale bars = 5 µm. **(D)** Quantitation showing the relative fluorescent intensity of CID per nucleus at prometaphase I. *n* = 50, 76, 57, 67. Error bars = SEM. **(E)** Representative images of early spermatid nuclei (T5 stage) stained with anti-CENP-C (green), anti-CID (magenta), and DAPI (blue) for each knockdown line and control (Rbp4-GAL4, CID RNAi, CAL1 RNAi, and CENP-C RNAi). CENP-C staining is absent, indicative of T5 stage spermatids. Scale bars = 3 µm. **(F)** Quantitation showing relative fluorescent intensity of CID per T5 nucleus. *n* = 39, 33, 32, 31. Error bars = SEM. **(G)** Quantitation showing the relative fluorescent intensity of CENP-C per nucleus at prometaphase I for each knockdown line and control (Rbp4-GAL4, CID RNAi, CAL1 RNAi, and CENP-C RNAi). *n* = 44, 39, 38, 39. Error bars = SEM. **(H)** Representative images of prometaphase I cells stained with anti-Spc105 (green) and anti-CID (magenta) antibodies and DAPI (blue) from the genetic lines Rbp4-GAL4 (control) and CENP-C RNAi. **(I)** Quantitation showing the relative fluorescent intensity of Spc105 per nucleus at prometaphase I. *n* = 45, 44. Error bars = SEM.

**Figure S4.**
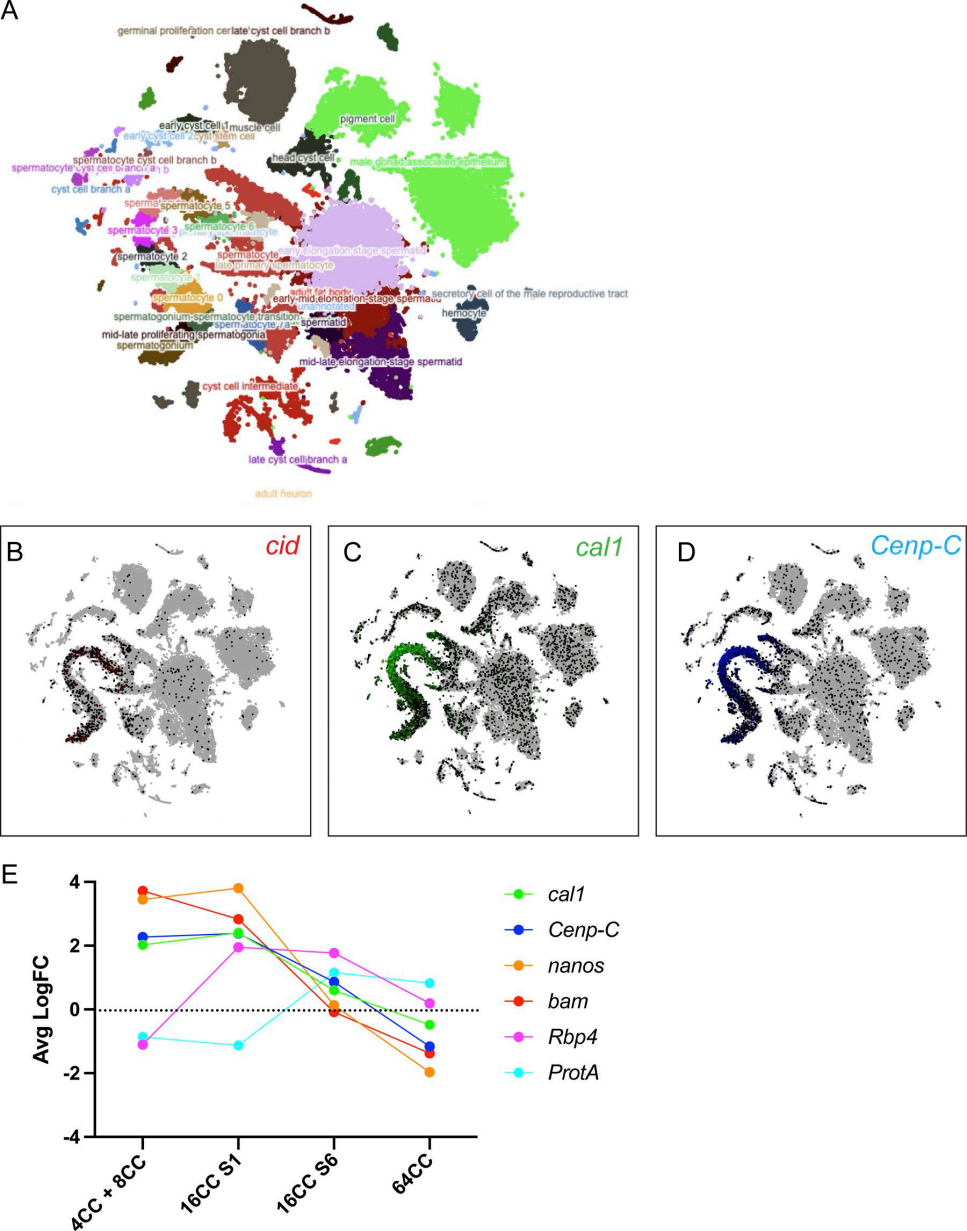
**Single-cell RNA-sequencing expression data for centromere proteins and known markers of spermatogenesis. (A)** UMAP graph displaying annotated testis cell clusters from the Fly Cell Atlas (Li et al., 2022), visualized using the SCope platform. **(B)** UMAP graph indicating expression levels of *cid* in adult testis. Black represents cells with lower expression, and red shows higher expression. **(C)** UMAP graph displaying *cal1* expression within the adult testis in green. **(D)***Cenp-C* expression within the adult testis is shown in blue. **(E)** Graph depicting the average log fold change of *cal1* and *Cenp-C*, along with several known markers of spermatogenesis, including *nanos* and *bam* (spermatogonia and spermatocytes), *Rbp4* (spermatocytes), and *Protamine A* (spermatocytes and spermatids). 4CC + 8CC data were derived from the mid/late-proliferating spermatogonia cluster in the 10× dataset. Spermatocyte 1 and 6 clusters were mapped to early and late prophase I using known expression patterns of *nanos*, *bam,* and *Rbp4*. Early/mid-elongation stage spermatids were used as the 64CC dataset. UMAP, Uniform Manifold Approximation and Projection.

To determine whether meiotic centromere deficiencies could be replenished during the second assembly event, CID levels in T5 spermatids were quantified after *cid*, *cal1*, and *Cenp-C* RNAi driven by *bam-GAL4* (Fig. 5, A and B). The *bam-GAL4* driver is expressed in 8CC and 16CC until late prophase I (S6). Transcription of most genes ceases in late prophase I of spermatogenesis, with few known exceptions (Vibranovski et al., 2010). Analysis of testis-specific single-cell RNA-sequencing data from the Fly Atlas (Li et al., 2022) project confirms that *bam, cid*, *cal1*, and *Cenp-C* are downregulated after late prophase I (16CC at S6) and remain so during spermatid differentiation (64CC) (Fig. S4, A–E), although some protein translation from preexisting transcripts is possible. Quantitation of centromeric CID in T5 spermatids confirmed that levels remain reduced (Fig. 5, A and B). CID levels were reduced by 53, 35, and 44% in the CID, CAL1, and CENP-C RNAi lines, respectively, and CENP-C was undetectable. This result indicates that the depletion of CID, CAL1, and CENP-C using the bam-GAL4 driver results in reduced CID at centromeres at prometaphase I, and that CID remains reduced in postmeiotic T5 spermatids. Interestingly, both CID and CENP-C RNAi showed a further significant reduction in CID from the levels previously established in prometaphase I (Fig. 2 G), whereas CAL1 RNAi did not. CID RNAi exhibited a further decrease in CID from 65% of control levels at prometaphase I to 47% in T5 early spermatids. CENP-C RNAi displayed the largest difference, reducing from 75% of control levels at prometaphase I to 56% after meiosis II. However, a very minimal reduction was observed in the CAL1 RNAi by comparison, from 70% of the prometaphase I control to 65% of the T5 stage control levels, suggesting it may not function during this second phase of assembly.

### CID protein synthesis and assembly are uncoupled during spermatogenesis

**Figure S5.**
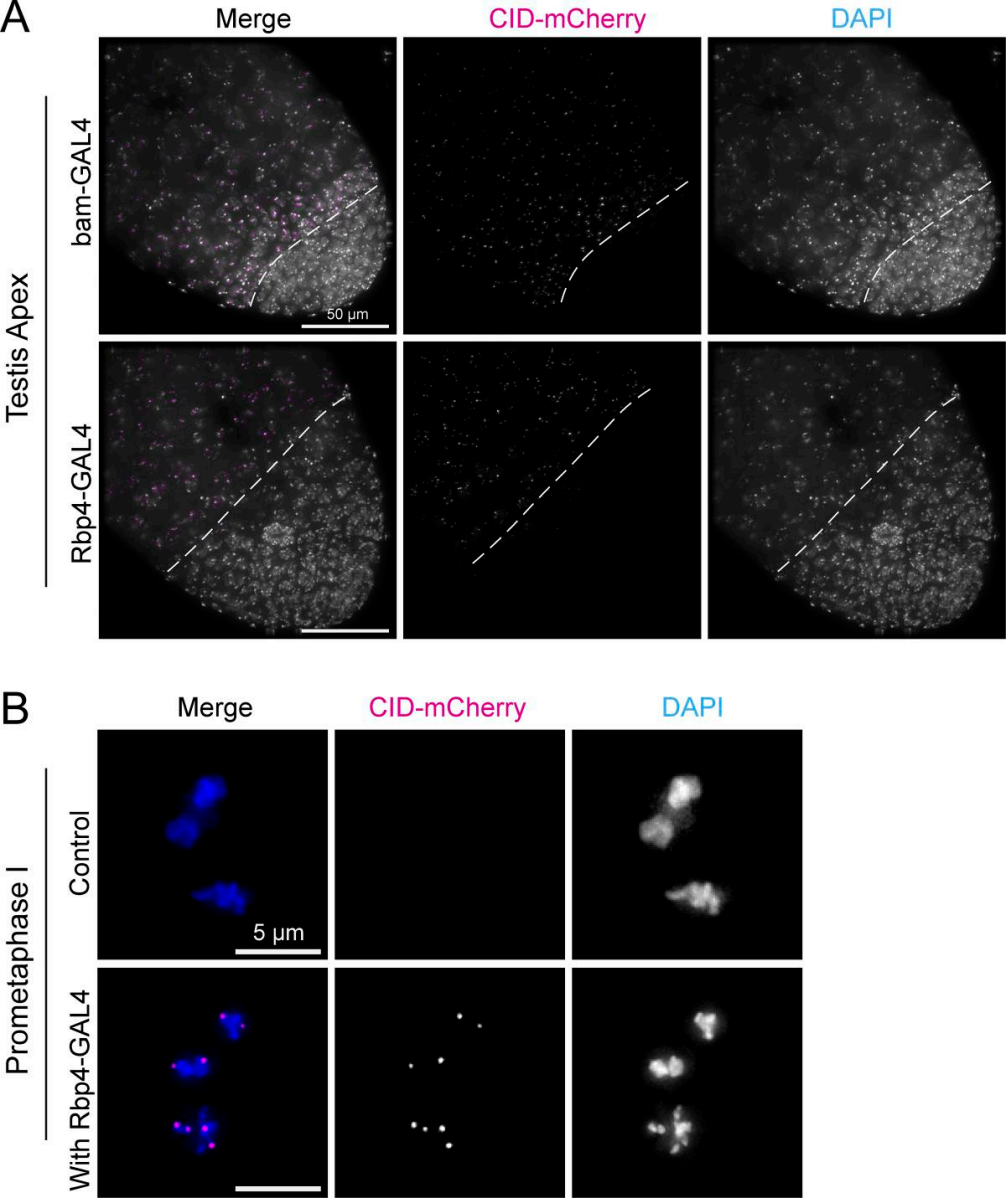
**Comparison of *bam-GAL4* and *Rbp4-GAL4* expression timing in *Drosophila* testis. (A)** Representative images of adult testis apices from crosses of either bam-GAL4 or Rbp4-GAL4 to the UAS-CID-mCherry fly line showing GAL4-driven expression of mCherry-tagged CID (magenta) and DAPI (blue). Scale bar = 50 µm. A white dashed line indicates the stage at which CID-mCherry signal starts. **(B)** Prometaphase I nuclei from pupal testes stained with DAPI (blue), showing Rbp4-GAL4–driven expression of the mCherry-tagged CID protein (magenta). The control comprised the UAS-CID-mCherry line without crossing to Rbp4-GAL4. Scale bar = 5 µm.

To gain increased temporal resolution for RNAi depletion studies in prophase I, a second GAL4 driver was adopted. *Rbp4-GAL4* expression begins at the S1 stage in 16CC spermatocytes (Fig. S4 E), providing a narrower window for RNAi induction (Butsch et al., 2023). The functionality of this driver was first tested by driving expression of mCherry-tagged CID under *UAS* control. As expected, CID-mCherry foci were first detectable at the S1-2 stage of early prophase I, notably later than *bam-GAL4*–driven expression, which appeared visible at 8CC (Fig. S5 A). At prometaphase I, CID-mCherry foci were visualized at centromeres, indicating that CID can be synthesized and assembled *de novo* at centromeres during prophase I (Fig. S5 B). Surprisingly, following *cid*, *cal1*, and *Cenp-C* knockdown using the *Rpb4-GAL4* driver, with respective depletions confirmed by qPCR (Fig. S2 B), quantitation of centromeric CID at prometaphase I did not reveal any change in CID intensity (Fig. 5, C and D). This result differs from the ∼30% reduction in CID that was measured in RNAi experiments using the *bam-GAL4* driver (Fig. 2 G). Thus, it appears that to significantly reduce levels of CID at the centromere during meiosis, RNAi constructs must be expressed in the prior cell cycle (8CC). However, in addition to timing differences, we cannot exclude that the amount of GAL4 expression differs between the drivers. Next, CID levels were quantified in T5 stage spermatids following *Rbp4-GAL4*–driven RNAi. Here, CID levels were reduced in both CID and CENP-C RNAi (Fig. 5, E and F), indicating that the CID protein translated in prophase I is assembled after meiosis II. Interestingly, no significant change in CID was detected in T5 spermatids in the CAL1 RNAi, suggesting it does not impact the second phase of assembly (Fig. 5, E and F). Taken together, we conclude that the CID protein synthesized during the 8-cell stage is loaded in prophase I (bam-GAL4 RNAi result), while the CID protein synthesized during the 16-cell stage is loaded following meiosis II (Rbp4-GAL4 RNAi result).

### Functionally distinct pools of CENP-C exist in prophase I to support CID assembly and kinetochore recruitment

To assess requirements for inner kinetochore recruitment specifically in prophase I, CENP-C levels in CID, CAL1, and CENP-C RNAi using the *Rbp4-GAL4* driver were quantified. As with centromeric CID levels (Fig. 5 D)*, Rbp4-GAL4*–driven CID and CAL1 RNAi did not significantly lower CENP-C levels at prometaphase I (Fig. 5 G). This resembles observations made with the *bam-GAL4* driver (Fig. 2, F and H) and further uncouples CID, CAL1, and CENP-C recruitment interdependencies in meiosis. *Rbp4-GAL4*–driven CENP-C RNAi did display reduced CENP-C levels (Fig. 5 G), confirming that the *Rbp4-GAL4* driver and RNAi pathway were active in this time window and capable of reducing available proteins for deposition during prophase I. Thus, improved temporal resolution provided by the *Rbp4-GAL4* driver enabled the separation of CENP-C function between early and late prophase I. Early in prophase I, CENP-C is susceptible to *bam-GAL4*–driven depletion and appears to function primarily in CID assembly (Fig. 2, F and G). Contrastingly, later in prophase I, CENP-C is susceptible to both *bam-GAL4–* and *Rbp4-GAL4–*driven RNAi and switches its function toward recruitment of kinetochore proteins. As a demonstration of this, *Rbp4-GAL4–*driven CENP-C RNAi caused a reduction in Spc105 recruitment, despite normal CID levels (Fig. 5, H and I). This provides further evidence that CENP-C recruitment dictates outer kinetochore assembly in late prophase I, separate from underlying CID.

### CAL1-independent CID assembly after meiosis II requires CENP-C

Unlike CID or CENP-C RNAi driven by *bam-GAL4*, CAL1 RNAi did not cause further reductions in CID levels at T5 after the second phase of assembly (Fig. 5, A and B). Together with the absence of CAL1 from centromeres beyond prophase I, this result raised questions regarding the role of CAL1 during the postmeiotic CID loading event. Centromeric CID levels were not impacted at prometaphase I in *Rbp4-GAL4*–driven CAL1 RNAi (Fig. 5, C and D). In fact, CID levels remained indistinguishable from those of the control even after the second phase of CID assembly in the early T5 spermatids (Fig. 5, E and F). This indicates that CID is loaded in a manner independent of its chaperone CAL1 after meiosis II. CENP-C disappears from centromeres in the window during which CID is assembled after meiosis II (Fig. S1 C). To establish whether CENP-C is required for this second phase of assembly, CID levels were quantified after *Rbp4-GAL4*–driven CENP-C RNAi. CENP-C levels were significantly reduced at prometaphase I, even as CID levels remained unchanged (Fig. 5 G). Moreover, CID was significantly reduced at centromeres in postmeiotic T5 stage spermatids, confirming that the pool of CENP-C synthesized later in prophase I is required for the second phase of CID assembly, as well as kinetochore formation at prometaphase I (Fig. 5, E and F). Taken together, these results demonstrate that CID assembly after meiosis II occurs independent of CAL1, but requires CENP-C.

## Discussion

Building on previous findings (Dunleavy et al., 2012; Raychaudhuri et al., 2012), we confirm and refine the requirements for two unique centromere assembly events in meiosis that ultimately ensure paternal centromeres harbor sufficient CID for embryogenesis. We separate assembly events occurring in early and late prophase I, with and without CAL1. In early prophase I, CAL1 functions in CID assembly together with CENP-C. At late prophase I, CAL1 is no longer detectable, and CID assembly is complete. At this point, CENP-C continues to be recruited to the centromere, but it is not critical for complete CID assembly. Rather, it functions in kinetochore recruitment. This pool of CENP-C also functions in the second phase of CID assembly on spermatids, which is, uniquely, independent of CAL1.

### Refining the requirements for CID assembly in prophase I

This study reveals a mutual dependency between CAL1 and CID in terms of stability and localization in early prophase I. At the S1 stage, CID depletion caused a dramatic reduction in CAL1, demonstrating that CAL1 requires newly synthesized non-nucleosomal CID for its stability and nuclear localization. Reciprocally, following CAL1 RNAi, centromeric CID levels were significantly reduced at S1. This suggests that CAL1 further functions to stabilize nucleosomal CID already incorporated at the centromere at this time. Similar observations were reported in mitotic cells, where CENP-A-HJURP and CID-CAL1 prenucleosomal complexes offer reciprocal stabilization in humans and flies, respectively (Erhardt et al., 2008). This is the first indication that similar mutual stabilization dynamics occur in meiosis.

We also demonstrate that in contrast to mitosis (Erhardt et al., 2008), CENP-C recruitment in meiosis is not entirely dependent on CID or CAL1 levels. According to the current mitotic model, CAL1 complexed with CID is initially recruited to the centromere by preexisting stably bound CENP-C. Newly localized CAL1 acts to recruit further CENP-C to the centromere, closing the epigenetic feedback loop (Roure et al., 2019). Here, we find that at the S1 stage, CENP-C can localize to centromeres in the absence of CAL1. Similarly, at prometaphase I, CENP-C can localize to centromeres at normal levels, despite lower levels of underlying CID. These results reveal that in meiosis, the recruitment of CENP-C by CID nucleosomes is not stoichiometric. The mechanism by which additional CENP-C is recruited to the centromere in the absence of a portion of CID nucleosomes is not understood, but could derive from the dimerization potential of CENP-C (Medina-Pritchard et al., 2020). Additionally, it is possible that under standard conditions, not every CID nucleosome recruits one CENP-C molecule. This permits a saturation of CID with CENP-C only when certain CID nucleosomes are lost, providing a mechanism by which CENP-C can be recruited to a level that prevents meiotic chromosome segregation defects. Such a mechanism might explain why Spc105 recruitment was enhanced in the CAL1 and CID RNAi despite a 30% reduction in CID.

### CID assembly after meiosis II is independent of CAL1, but requires CENP-C

This study establishes for the first time the requirements for the postmeiotic phase of CID assembly. CAL1 cannot be visualized at the centromere from mid-prophase I, remains undetectable for the rest of spermatogenesis, and is absent from mature sperm (Raychaudhuri et al., 2012). Here, we show that CAL1 depletion does not specifically impact CID levels in T5 spermatids, indicating that CID deposition occurs normally despite the absence of its chaperone. This represents the first known example of a CAL1-independent deposition event in flies, or indeed any species. It is interesting to speculate whether such an assembly could occur independently of HJURP in mammals. Furthermore, we show that this second phase of CID assembly is dependent on CENP-C. No evidence exists that CENP-C can assemble CID nucleosomes directly or that CID is capable of self-assembly; thus, we suggest that CENP-C is required to recruit unknown or canonical histone assembly factors for this purpose. The function of this second phase of deposition may be to prime spermatid cells for the drastic chromatin remodeling event involved with protamine exchange, during which CID remains one of few histones retained on the mature sperm (Raychaudhuri et al., 2012). Indeed, our analysis of the overall CID dynamics in the germline indicates that the level of CID on T5 spermatids is twofold higher than expected for a haploid cell. Precisely how *Drosophila* CID is maintained on mature sperm in the absence of both CENP-C and CAL1 requires further investigation.

### Functionally distinct pools of meiotic CENP-C are required for CID assembly and kinetochore recruitment

We also identify distinct pools of CENP-C with separate, nonoverlapping functions in meiosis. In early prophase, one CENP-C pool functions in CID assembly, presumably in the recruitment of CAL1. At prometaphase I, another CENP-C pool assembles the kinetochore. Furthermore, this pool of CENP-C does not function in CID assembly at that time. It is likely that CENP-C preferentially functions in kinetochore recruitment later in prophase I because CAL1 is absent. We show that a minor pool of CENP-C loaded at prometaphase I is critical for a large proportion of Spc105 recruitment, which in turn is crucial for meiotic chromosome segregation. These findings parallel observations in *Drosophila* females in which a pool of CENP-C maintains centromeric CID in oocytes, which can be exchanged for a new and functionally distinct pool of CENP-C in prophase I (Fellmeth et al., 2023). Remarkably, in males, this “kinetochore” pool of CENP-C takes on an additional function in postmeiosis II, where it is required for CID assembly on spermatids. This deposition event occurs in the absence of CAL1, and it is possible that CENP-C functions in the recruitment of a novel CID chaperone and assembly factor.

### Synthesis and deposition of the CID protein are uncoupled during spermatogenesis

In mitosis, CID/CENP-A is primarily synthesized in the G2 phase and deposited at the end of mitosis in the same cell cycle (Jansen et al., 2007; Schuh et al., 2007; Mellone et al., 2011). Here, we identify that the timing of CID synthesis and its cell cycle assembly are uncoupled during spermatogenesis. For the first phase of assembly in meiotic prophase I, most of the newly assembled CID protein appears to be synthesized in the preceding mitotic division. The temporal resolution of RNAi depletion experiments allowed us to conclude that the pool of CID assembled during prophase I is transcribed and translated at the 8CC stage. We note that if excess CID is provided in prophase I (via CID-mCherry overexpression driven by the *Rbp4-GAL4* driver), it can load at the centromere. However, under normal expression conditions, we propose that the CID pool synthesized at the 8CC stage is preferentially loaded. For the second phase of assembly in postmeiotic spermatids, we found that the newly assembled CID protein is synthesized in meiotic prophase I. Exactly how soluble CID protein is chaperoned to survive both meiotic divisions is not known, but it is unlikely to work via CAL1, which is undetectable at this stage. This is an intriguing example of a protein synthesized in one cell cycle and used in the next, possibly facilitated by incomplete cytokinesis occurring in germline cysts. Comparable to most species, *Drosophila* spermatocytes undergo successive meiotic divisions with a very short intervening interkinesis (Fuller, 1998; Giansanti and Fuller, 2012). The lack of proper gap phases, during which the CID protein is normally synthesized, may necessitate pretranslation of any protein required shortly after the second division.

### Conserved CID and CENP-C dynamics in the male and female germline

This work highlights a critical function for CENP-C recruitment in male meiosis. Strikingly, despite an ∼50% reduction in CID in T5 spermatids and only 10% of spermatids displaying a normal karyotype in CENP-C RNAi, we did not measure any defect in male fertility (Fig. 4 G). This result is surprising, though it likely reflects the amplificatory nature of spermatogenesis; each GSC gives rise to a cyst of 64 spermatids, with multiple cysts in each testis. Therefore, despite high rates of meiotic chromosome missegregation, one normal sperm manages to fertilize the egg. To offset the 50% reduction in CID, it is possible that there is a compensatory loading of CID on paternal chromosomes in the embryo, which is proposed to occur in CENP-C–depleted mouse oocytes (Tower et al., 2025). *Drosophila* male meiosis is atypical, lacking the signature features of meiosis of synapsis and recombination (Cenci et al., 1994), and therefore might not reflect centromere dynamics in meiosis in general. However, our results align well with the dynamics of CENP-C reported in female mice and *Drosophila*, which exhibit the classical features of meiosis (Smoak et al., 2016; Fellmeth et al., 2023). In female flies, it was proposed that replenishing the pool of CENP-C is necessary due to the extended arrest in prophase I that occurs in oocytes (Fellmeth et al., 2023). Yet, male meiosis does not encompass a prophase I arrest in any species. Therefore, mechanisms of CID assembly and CENP-C turnover are more likely to be related to a general switch in CENP-C function at prometaphase I that is critical for both sexes.

## Materials and methods

### *Drosophila* stocks and husbandry

Fly stocks were propagated at 25°C on Nutri-Fly standard medium supplemented with 0.5% propionic acid and 1% nipagin under a 12-h light–dark cycle. These stocks were obtained from various sources as documented here: from Vienna *Drosophila* Research Centre (VDRC), *UAS-cid lhRNAi* (#102090: CID RNAi), *UAS-cal1 lhRNAi* (#45248: CAL1 RNAi), *UAS-Cenp-C lhRNAi* (#33790, discontinued, but exists with the same construct ID 10208 as #33792: CENP-C RNAi); and from Bloomington *Drosophila* Stock Centre (BDSC), wild-type Oregon R (#25211), *y*^*1*^w* P{w^*+mC*^*= bam-GAL4:VP16}1* (#8057: bam-GAL4), w*; PBac{y^*+mDint2*^*w*^*+mC*^*= Rbp4-GAL4.B}VK00027* (#600281: Rbp4-GAL4). The fly line *bam-GAL4; Tubulin-GFP* was generated in the lab by a series of crosses between BDSC #80579 and Kyoto Stock Center #109603. From various other sources, the overexpression lines *UAS-cid-mCherry* (Dattoli et al., 2020), *UASp-HA-Cenp-C/TM6 Tb* (originally gifted as *UASp-HA-Cenp-C/TM3 Sb* from Kim McKim, Rutgers, The State University of New Jersey, Piscataway, New Jersey, United States of America), and the CENP-C rescue line *UASp HA-Cenp-C; UAS-Cenp-C lhRNAi* (Carty et al., 2021) were obtained. To perform an experiment, virgin females from the specific *GAL4* driver lines were mated with males displaying the appropriate markers from the RNAi or overexpression responder lines and the progeny were let develop at 29°C. F_1_ progeny were dissected at the early pupal stage of development to enrich for meiotic cells and early spermatid stages. Three biological replicates were performed for each experiment.

### *Drosophila* testis tissue preparation

Testes (adult and pupal) were dissected in 1X PBS and fixed on a SuperFrost slide in 4% paraformaldehyde (diluted with 1X PBS) for 10 min. All common reagents were obtained from Thermo Fisher Scientific or Merck unless otherwise specified. After fixation, the samples were gently squashed beneath a hydrophobic RainX-treated coverslip and snap-frozen in liquid nitrogen. The coverslip was quickly removed, and the samples were stored in 70% ethanol at −20°C until further processing. Processing for immunofluorescence and fluorescence *in situ* hybridization involved passing through an ice-cold ethanol series (2 min each in 75, 85, and 95% ethanol), allowing the samples to air-dry for ∼2 min and to permeabilize for 10 min in 1X PBS/0.4% Triton X-100 (PBST 0.4%) at room temperature.

### Quantitative PCR

Total RNA was isolated from 45 to 55 testes of P4−P5 pupae using TRI reagent (Sigma-Aldrich) and chloroform (Sigma-Aldrich), followed by storage in 75% ethanol at −20°C until further processing. The RNA was then resuspended in nuclease-free water and treated with DNase I, Amplification Grade (Invitrogen), to remove any traces of DNA. Complementary DNA (cDNA) was synthesized from 1.1 µg of RNA using RevertAid First Strand cDNA Synthesis Kit (Invitrogen) and used in qPCR with Luna Universal qPCR Master Mix (New England Biolabs). The qPCR was run in 0.1 ml MicroAmp Fast Optical 96-Well Reaction Plates (Applied Biosystems) sealed with Optical Adhesive Covers (Applied Biosystems), on QuantStudio 5 Real-Time PCR System (Applied Biosystems). Three technical replicates per each of two or three biological replicates of each experiment were carried out. The following primers were used (Table 1).

**Table 1.** Primer sequences and efficiencies for qPCR

Gene	Primer	Sequence	Average efficiency	Source
*αTub84B*	Forward	5′-TGT​CGC​GTG​TGA​AAC​ACT​TC-3′	94.4%	Ponton et al. (2011)
	Reverse	5′-AGC​AGG​CGT​TTC​CAA​TCT​G-3′		
*eEF1α2*	Forward	5′-GCG​TGG​GTT​TGT​GAT​CAG​TT-3′	99.3%	
	Reverse	5′-GAT​CTT​CTC​CTT​GCC​CAT​CC-3′		
*cid*	Forward	5′-GAA​GAC​GGC​ACC​GAC​TAC​GG-3′	95.5%	Collins et al. (2018)
	Reverse	5′-CGT​CGA​GGA​ACG​CCG​ATT​GT-3′		
*Cenp-C*	Forward	5′-AGG​AGA​CCG​TAA​ACT​TGA​CCC​G-3′	97.7%	​
	Reverse	5′-TTC​TCT​GTG​CAA​GGT​GTG​CTG-3′		
*cal1*	Forward	5′-AAT​GAG​GAT​AAC​GAG​GCA​AAG-3′	97.8%	​
	Reverse	5′-TGT​CCA​GAA​TGC​GAT​CCA​G-3′		

The efficiency of primers was evaluated based on mean Ct of standards containing the mixture of all cDNA samples using NEBioCalculator (v.1.17.4). Relative fold change was calculated in relation to the average of *bam-GAL4* control experiments for each gene as follows:

(EfficiencyGOI^∆CtGOL)/GeoMean(EfficiencyREF^∆CtREF)

where GOI is the gene of interest, REF is the reference gene, and GeoMean is the geometric mean of the values of the two reference genes. Statistical analysis of the amplification data included the Shapiro–Wilk normality test and one-way ANOVA followed by Tukey’s HSD test.

### Immunofluorescence

Samples were blocked with 1% bovine serum albumin in 1X PBS for 1 h at room temperature. Primary antibodies were diluted in blocking buffer and incubated with the samples overnight at 4°C. The next day, the samples were washed three times for 10 min in 1X PBST 0.4% and incubated with secondary antibodies (1:500 dilution in blocking buffer) for 1 h at room temperature in the dark. Then, a further 3 × 10-min washes in PBST 0.4% were performed. Samples were treated with DAPI (1 μg/ml in 1X PBS) for 5 min at room temperature and washed in 1X PBS for 10 min. Finally, they were mounted in SlowFade Gold Antifade Reagent (Invitrogen) and stored at −20°C.

### Antibodies

Primary antibodies used for immunofluorescence include rat anti-CID (#61735; Active Motif; used at 1:500), sheep anti-CENP-C (Dattoli et al., 2020; used at 1:500), sheep anti-CAL1 (Kochendoerfer et al., 2023; used at 1:1,000), sheep anti-Spc105 (gifted from Przewloka et al. [2011]; used at 1:500), and mouse anti-HA (#26183; Thermo Fisher Scientific; 1:500). Species-specific Alexa Fluor 488, 546, 555, and 647 secondary antibodies were used at a standard 1:500 dilution: goat anti-rat Alexa Fluor 546 (A11081; Invitrogen), donkey anti-rat Alexa Fluor 555 (A78945; Invitrogen), donkey anti-rat Alexa Fluor 647 (A48272; Invitrogen), donkey anti-sheep Alexa Fluor 488 (ab150177; Abcam), donkey anti-sheep Alexa Fluor 647 (A21448; Invitrogen), donkey anti-mouse Alexa Fluor 546 (A10036; Invitrogen).

### Fluorescence *in situ* hybridization

To investigate homologous chromosome and sister chromatid segregation during meiosis I and II, fluorescently labeled probes for the X chromosome (359 bp repeat), Y chromosome (AATAC)_6_, and second (Responder), third (Dodeca satellite), and fourth chromosome (AATAT)_6_ were used. Probes were synthesized as single-stranded oligonucleotides conjugated with 5′ ATTO fluorophores by Eurofins using sequences obtained from Tsai et al. (2011) and Ferree and Barbash (2009). Samples were washed three times for 10 min in 2X saline–sodium citrate–Tween 0.1% (2X SSC-Tw 0.1%) at room temperature, followed by one 10-min wash in 2X SSC-Tw 0.1% + 25% formamide and 10-min wash in 2X SSC-Tw 0.1% + 50% formamide at room temperature. Prehybridization was carried out in 2X SSC-Tw 0.1% + 50% formamide at 37°C for 2 h. A quantity of 20 ng was used per sample for the second, third, X, and Y chromosome probes, and 40 ng was used for the fourth chromosome probe. The probes were added to 1X hybridization buffer (3X SCC, 10 % dextran sulfate) with 50% formamide. 20 μl of hybridization buffer containing the respective FISH probes was added to each coverslip. Samples were inverted over the coverslips and sealed with Marabu Fixogum rubber cement. Denaturation was completed at 90°C for 4 min and followed with incubation overnight in a humidified chamber at 20°C. The coverslip and rubber cement were removed, and a series of posthybridization washes were completed as follows: 1 × 10-min wash at 20°C in 2X SSC-Tw + 50% formamide, 2 × 30-min washes at 20°C in 2X SSC-Tw 0.1% + 50% formamide, 1 × 10-min wash in 2X SSC-Tw 0.1% + 25% formamide at room temperature,, and 3 × 10-min washes in 2X SSC-Tw 0.1% at room temperature. Samples were treated with DAPI for 5 min, washed in 1X PBS for 10 min, mounted in SlowFade Gold Antifade Reagent, and stored at −20°C.

### Microscopy and image analysis

Experiments were imaged at 25°C with the Delta Vision Elite microscope (Applied Precision, Imsol) using the 40× and 60× oil immersion Olympus X-line UPLXAPO 40X 1.4NA and 1.42NA objectives and CoolSNAP_HQ2/HQ2-ICX285 camera. Z-stacks of 0.2 μm step size were used. Fluorescence was passed through a 430- to 455-nm, 490- to 540-nm, 575- to 620-nm, and 655- to 755-nm band-pass filter for detection for DAPI and Alexa Fluor 488, 546, and 647, respectively. Exposure times and transmission percentages were kept constant through each experiment. Images acquired at the microscope were deconvolved (conservative approach) using softWoRx application (Applied Precision, Imsol) and processed in FIJI/ImageJ.

### Image analysis

To quantify protein levels in the samples, fluorescent intensity was measured using FIJI/ImageJ. Single nuclei were isolated among the images (8-bit) and Z-projected with maximal intensity, ensuring to capture the entirety of the centromeric signal. All images were saved as .tiff files. The background was subtracted (rolling ball radius set to 50.0) from the projected images, and thresholding using the default algorithm was applied to select the entirety of the centromeric signal. Integrated density (mean gray value x area) was summed to generate the total fluorescent intensity per nucleus. Statistical analyses were performed using GraphPad Prism software. Each sample population was tested for normality using a Shapiro–Wilk test, and corresponding parametric or nonparametric statistical tests were performed accordingly (Welch’s *t* test or Mann–Whitney U test). *P* values are displayed on each graph.

### Fertility assays

One virgin wild-type female was crossed to one RNAi male at 29°C, and the total number of offspring per wild-type female was scored after 14 days.

### Online Supplemental material

Fig. S1 shows the localization dynamics of the centromere proteins CAL1 and CENP-C at key stages of spermatogenesis. Fig. S2 shows qPCR validation of RNAi for *cid*, *Cenp-C*, and *cal1* in each RNAi fly line. Fig. S3 shows the percentage chromosome loss/aneuploidy of the autosomes and sex chromosomes after meiosis II in each RNAi line (CID RNAi, CAL1 RNAi, and CENP-C RNAi). Fig. S4 shows single-cell RNA-sequencing expression data for *cid*, *cal1*, and *Cenp-C* from the Fly Cell Atlas (Li et al., 2022). Fig. S5 shows the difference in expression timing between *bam-GAL4* and *Rbp4-GAL4*, as well as *Rbp4-GAL4*–driven expression of mCherry-tagged CID during the first phase of meiotic CID assembly.

## Supplementary Material

Review History

## Data Availability

All data supporting the findings of this study are included in the published article and its supplemental material, or are available from the corresponding author upon reasonable request.

## References

[bib1] Adams, E.E., Q. He, and B.D. McKee. 2020. How noncrossover homologs are conjoined and segregated in *Drosophila* male meiosis I: Stable but reversible homolog linkers require a novel separase target protein. PLoS Genet. 16:e1008997. 10.1371/journal.pgen.1008997 33002007 PMC7529219

[bib2] Allshire, R.C., and G.H. Karpen. 2008. Epigenetic regulation of centromeric chromatin: Old dogs, new tricks? Nat. Rev. Genet. 9:923–937. 10.1038/NRG2466 19002142 PMC2586333

[bib3] Butsch, T.J., A.E. Johnson, and K.A. Bohnert. 2023. Rbp4-Gal4, a germline driver that activates in meiosis, reveals functions for VCP in spermatid development. Fly. 17:2234795. 10.1080/19336934.2023.2234795 37436409 PMC10339771

[bib44] Carty, B.L., A.A. Dattoli, and E.M. Dunleavy. 2021. CENP-C functions in centromere assembly, the maintenance of CENP-A asymmetry and epigenetic age in Drosophila germline stem cells. PLoS Genet. 17:e1009247. 10.1371/journal.pgen.1009247 34014920 PMC8136707

[bib4] Cenci, G., S. Bonaccorsi, C. Pisano, F. Verni, and M. Gatti. 1994. Chromatin and microtubule organization during premeiotic, meiotic and early postmeiotic stages of *Drosophila* melanogaster spermatogenesis. J. Cell Sci. 107:3521–3534. 10.1242/JCS.107.12.3521 7706403

[bib5] Chen, C.-C., M.L. Dechassa, E. Bettini, M.B. Ledoux, C. Belisario, P. Heun, K. Luger, and B.G. Mellone. 2014. CAL1 is the *Drosophila* CENP-A assembly factor. J. Cell Biol. 204:313–329. 10.1083/jcb.201305036 24469636 PMC3912524

[bib6] Collins, C.M., B. Malacrida, C. Burke, P.A. Kiely, and E.M. Dunleavy. 2018. ATP synthase F1 subunits recruited to centromeres by CENP-A are required for male meiosis. Nat. Commun. 9:2702. 10.1038/s41467-018-05093-9 30006572 PMC6045659

[bib43] Dattoli, A.A., B.L. Carty, A.M. Kochendoerfer, C. Morgan, A.E. Walshe, and E.M. Dunleavy. 2020. Asymmetric assembly of centromeres epigenetically regulates stem cell fate. J. Cell Biol. 219:e201910084. 10.1083/jcb.201910084 32328637 PMC7147107

[bib7] Dubruille, R., B. Horard, and B. Loppin. 2025. A haystack in the needle: Packaging sperm DNA in insects. Curr. Opin. Genet. Dev. 93:102378. 10.1016/j.gde.2025.102378 40627886

[bib8] Dunleavy, E.M., N.L. Beier, W. Gorgescu, J. Tang, S.V. Costes, and G.H. Karpen. 2012. The cell cycle timing of centromeric chromatin assembly in *Drosophila* meiosis is distinct from mitosis yet requires CAL1 and CENP-C. PLoS Biol. 10:e1001460. 10.1371/JOURNAL.PBIO.1001460 23300382 PMC3531500

[bib9] Erhardt, S., B.G. Mellone, C.M. Betts, W. Zhang, G.H. Karpen, and A.F. Straight. 2008. Genome-wide analysis reveals a cell cycle-dependent mechanism controlling centromere propagation. J. Cell Biol. 183:805–818. 10.1083/jcb.200806038 19047461 PMC2592830

[bib10] Fellmeth, J.E., J.K. Jang, M. Persaud, H. Sturm, N. Changela, A. Parikh, and K.S. McKim. 2023. A dynamic population of prophase CENP-C is required for meiotic chromosome segregation. PLoS Genet. 19:e1011066. 10.1371/journal.pgen.1011066 38019881 PMC10721191

[bib45] Ferree, P.M., and D.A. Barbash. 2009. Species-specific heterochromatin prevents mitotic chromosome segregation to cause hybrid lethality in Drosophila. PLoS Biol. 7:e1000234. 10.1371/journal.pbio.1000234 19859525 PMC2760206

[bib11] Foltz, D.R., L.E.T. Jansen, B.E. Black, A.O. Bailey, J.R. Yates Iii, and D.W. Cleveland. 2006. The human CENP-A centromeric nucleosome-associated complex. Nat. Cell Biol. 8:458–469. 10.1038/ncb1397 16622419

[bib12] Fukagawa, T., and W.C. Earnshaw. 2014. The centromere: Chromatin foundation for the kinetochore machinery. Dev. Cell. 30:496–508. 10.1016/J.DEVCEL.2014.08.016 25203206 PMC4160344

[bib13] Fuller, M.T. 1998. Genetic control of cell proliferation and differentiation in *Drosophila* spermatogenesis. Semin. Cell Dev. Biol. 9:433–444. 10.1006/SCDB.1998.0227 9813190

[bib14] Giansanti, M.G., and M.T. Fuller. 2012. What *Drosophila* spermatocytes tell us about the mechanisms underlying cytokinesis. Cytoskeleton (Hoboken). 69:869–881. 10.1002/cm.21063 22927345 PMC4165571

[bib15] Jansen, L.E.T., B.E. Black, D.R. Foltz, and D.W. Cleveland. 2007. Propagation of centromeric chromatin requires exit from mitosis. J. Cell Biol. 176:795–805. 10.1083/JCB.200701066 17339380 PMC2064054

[bib16] Joshi, J.N., N. Changela, L. Mahal, J. Jang, T. Defosse, L.I. Wang, A. Das, J.G. Shapiro, and K. McKim. 2024. Meiosis-specific functions of kinetochore protein SPC105R required for chromosome segregation in *Drosophila* oocytes. Mol. Biol. Cell. 35:ar105. 10.1091/MBC.E24-02-0067 38865189 PMC11321039

[bib17] Kixmoeller, K., P.K. Allu, and B.E. Black. 2020. The centromere comes into focus: From CENP-A nucleosomes to kinetochore connections with the spindle. Open Biol. 10:200051. 10.1098/RSOB.200051 32516549 PMC7333888

[bib18] Kochendoerfer, A.M., R.S. Keegan, and E.M. Dunleavy. 2023. Centromere proteins are asymmetrically distributed between newly divided germline stem and daughter cells and maintain a balanced niche in *Drosophila* males. Mol. Biol. Cell. 34:ar42. 10.1091/mbc.E22-10-0466 36920070 PMC10162413

[bib19] Kwenda, L., C.M. Collins, A.A. Dattoli, and E.M. Dunleavy. 2016. Nucleolar activity and CENP-C regulate CENP-A and CAL1 availability for centromere assembly in meiosis. Development. 143:1400–1412. 10.1242/DEV.130625/-/DC1 27095496 PMC4852514

[bib20] Kyriacou, E., and P. Heun. 2023. Centromere structure and function: Lessons from *Drosophila*. Genetics. 225:iyad170. 10.1093/GENETICS/IYAD170 37931172 PMC10697814

[bib21] Li, H., J. Janssens, M. de Waegeneer, S.S. Kolluru, K. Davie, V. Gardeux, W. Saelens, F.P.A. David, M. Brbić, K. Spanier, . 2022. Fly cell atlas: A single-nucleus transcriptomic atlas of the adult fruit fly. Science. 375:eabk2432. 10.1126/science.abk2432 PMC8944923 35239393

[bib22] McKee, B.D., L. Habera, and J.A. Vrana. 1992. Evidence that intergenic spacer repeats of *Drosophila* melanogaster Rrna genes function as X-Y pairing sites in Male meiosis, and a general model for achiasmatic pairing. Genetics. 132:529–544. 10.1093/GENETICS/132.2.529 1330825 PMC1205154

[bib23] McKinley, K.L., and I.M. Cheeseman. 2016. The molecular basis for centromere identity and function. Nat. Rev. Mol. Cell Biol. 17:16–29. 10.1038/NRM.2015.5 26601620 PMC8603311

[bib24] Medina-Pritchard, B., V. Lazou, J. Zou, O. Byron, M.A. Abad, J. Rappsilber, P. Heun, and A.A. Jeyaprakash. 2020. Structural basis for centromere maintenance by *Drosophila* CENP-A chaperone CAL1. EMBO J. 39:e103234. 10.15252/EMBJ.2019103234 32134144 PMC7110144

[bib25] Mellone, B.G., K.J. Grive, V. Shteyn, S.R. Bowers, I. Oderberg, and G.H. Karpen. 2011. Assembly of *Drosophila* centromeric chromatin proteins during mitosis. PLoS Genet. 7:e1002068. 10.1371/journal.pgen.1002068 PMC3093364 21589899

[bib26] Monen, J., P.S. Maddox, F. Hyndman, K. Oegema, and A. Desai. 2005. Differential role of CENP-A in the segregation of holocentric C. elegans chromosomes during meiosis and mitosis. Nat. Cell Biol. 7:1248–1255. 10.1038/ncb1331 16273096

[bib27] Okada, M., I.M. Cheeseman, T. Hori, K. Okawa, I.X. Mcleod, J.R. Yates Iii, A. Desai, and T. Fukagawa. 2006. The CENP-H-I complex is required for the efficient incorporation of newly synthesized CENP-A into centromeres. Nat. Cell Biol. 8:446–457. 10.1038/ncb1396 16622420

[bib28] Ponton, F., M.P. Chapuis, M. Pernice, G.A. Sword, and S.J. Simpson. 2011. Evaluation of potential reference genes for reverse transcription-qPCR studies of physiological responses in *Drosophila* melanogaster. J. Insect Physiol. 57:840–850. 10.1016/J.JINSPHYS.2011.03.014 21435341

[bib29] Przewloka, M.R., Z. Venkei, V.M. Bolanos-Garcia, J. Debski, M. Dadlez, and D.M. Glover. 2011. CENP-C is a structural platform for kinetochore assembly. Curr. Biol. 21:399–405. 10.1016/J.CUB.2011.02.005 21353555

[bib30] Ranjan, R., J. Snedeker, and X. Chen. 2019. Asymmetric centromeres differentially coordinate with mitotic machinery to ensure biased sister chromatid segregation in germline stem cells. Cell Stem Cell. 25:666–681.e5. 10.1016/J.STEM.2019.08.014 31564548 PMC6842444

[bib31] Raychaudhuri, N., R. Dubruille, G.A. Orsi, H.C. Bagheri, B. Loppin, and C.F. Lehner. 2012. Transgenerational propagation and quantitative maintenance of paternal centromeres depends on Cid/Cenp-A presence in *Drosophila* sperm. PLoS Biol. 10.e1001434. 10.1371/JOURNAL.PBIO.1001434 23300376 PMC3531477

[bib32] Roure, V., B. Medina-Pritchard, V. Lazou, L. Rago, E. Anselm, D. Venegas, A.A. Jeyaprakash, and P. Heun. 2019. Reconstituting *Drosophila* centromere identity in human cells. Cell Rep. 29:464–479.e5. 10.1016/J.CELREP.2019.08.067 31597104 PMC6900781

[bib33] Rowley, G., and L.E.T. Jansen. 2025. A brief historical perspective on cell cycle control of CENP-A assembly and inheritance. Chromosome Res. 33:15. 10.1007/S10577-025-09774-2 40715876 PMC12296988

[bib34] Schittenhelm, R.B., F. Althoff, S. Heidmann, and C.F. Lehner. 2010. Detrimental incorporation of excess Cenp-A/Cid and Cenp-C into *Drosophila* centromeres is prevented by limiting amounts of the bridging factor Cal1. J. Cell Sci. 123:3768–3779. 10.1242/jcs.067934 20940262

[bib35] Schuh, M., C.F. Lehner, and S. Heidmann. 2007. Incorporation of *Drosophila* CID/CENP-A and CENP-C into centromeres during early embryonic anaphase. Curr. Biol. 17:237–243. 10.1016/J.CUB.2006.11.051 17222555

[bib36] Smoak, E.M., P. Stein, R.M. Schultz, M.A. Lampson, and B.E. Black. 2016. Long-term retention of CENP-A nucleosomes in mammalian oocytes underpins transgenerational inheritance of centromere identity. Curr. Biol. 26:1110–1116. 10.1016/J.CUB.2016.02.061 27040782 PMC4846481

[bib37] Štiavnická, M., A. Ní Nualláin, C.M. Collins, and E.M. Dunleavy. 2025. CENP-A is diluted during bovine spermatogenesis and is maintained at internally positioned centromere clusters in mature bull sperm. Chromosome Res. 33:20. 10.1007/S10577-025-09781-3 40958016 PMC12441075

[bib38] Sundararajan, K., and A.F. Straight. 2022. Centromere identity and the regulation of chromosome segregation. Front. Cell Dev. Biol. 10:914249. 10.3389/FCELL.2022.914249 35721504 PMC9203049

[bib39] Swartz, S.Z., L.S. McKay, K.C. Su, L. Bury, A. Padeganeh, P.S. Maddox, K.A. Knouse, and I.M. Cheeseman. 2019. Quiescent cells actively replenish CENP-A nucleosomes to maintain centromere identity and proliferative potential. Dev. Cell. 51:35–48.e7. 10.1016/J.DEVCEL.2019.07.016 31422918 PMC6783363

[bib40] Tower, C.A., G. Manske, E.L. Ferrell, D.N. Anbarci, K. Jorgensen, B. Ma, M. Aboelenain, R. Ranjan, S. Chakraborty, L. Moritz, . 2025. Maternal CENP-C restores centromere symmetry in mammalian zygotes to ensure proper chromosome segregation. Dev. Cell. 61:146–163.e10. 10.1016/j.devcel.2025.08.017 40997799 PMC12479092

[bib41] Tsai, J.-H., R. Yan, and B.D. McKee. 2011. Homolog pairing and sister chromatid cohesion in heterochromatin in *Drosophila* male meiosis I. Chromosoma. 120:335–351. 10.1007/S00412-011-0314-0 21384262

[bib42] Vibranovski, M.D., D.S. Chalopin, H.F. Lopes, M. Long, and T.L. Karr. 2010. Direct evidence for postmeiotic transcription during *Drosophila* melanogaster spermatogenesis. Genetics. 186:431–433. 10.1534/GENETICS.110.118919 20610406 PMC2940308

