# Supplementary material for: Meiotic CENP-C supports centromere assembly and kinetochore recruitment in spermatogenesis
Source: J Cell Biol. 2026 Jun 30;225(9):e202512053. doi: 10.1083/jcb.202512053 (PMC13317442; doi:10.1083/jcb.202512053)
Supplement: Review History [file jcb_202512053_review_history.pdf]

JCB

Meiotic CENP-C supports centromere assembly and kinetochore recruitment in spermatogenesis

Rachel Keegan, Dina Malkeyeva, Meg Weever, and Elaine Dunleavy

Corresponding Author(s): Elaine Dunleavy, Ollscoil na Gaillimhe - University of Galway

Review Timeline:

Submission Date:	2025-12-05
Editorial Decision:	2026-02-17
Revision Received:	2026-05-11
Editorial Decision:	2026-05-26
Revision Received:	2026-06-05

Monitoring Editor: Arshad Desai

Scientific Editor: Dan Simon

Transaction Report:

(Note: With the exception of the correction of typographical or spelling errors that could be a source of ambiguity, letters and reports are not edited. The original formatting of letters and referee reports may not be reflected in this compilation.)

DOI: https://doi.org/10.1083/jcb.202512053

February 17, 2026

Re: JCB manuscript #202512053

Elaine Dunleavy
Ollscoil na Gaillimhe - University of Galway

Dear Dr. Dunleavy,

Thank you for submitting your manuscript entitled "Distinct pools of CENP-A and CENP-C support unique phases of centromere assembly in spermatogenesis." The manuscript was assessed by expert reviewers, whose comments are appended to this letter. We invite you to submit a revision if you can address the reviewers' key concerns, as outlined here.

Based on the reviewer feedback, we believe it will be critical to address the issue of RNAi efficiency. We also encourage any efforts to distinguish between newly loaded and pre-existing pools but appreciate that this many not be technically feasible and will not require it for a revision.

While you are revising your manuscript, please also attend to the following editorial points to help expedite the publication of your manuscript. Please direct any editorial questions to the journal office.

GENERAL GUIDELINES:

Text limits: Character count for an Article is < 40,000, not including spaces. Count includes title page, abstract, introduction, results, discussion, and acknowledgments. Count does not include materials and methods, figure legends, references, tables, or supplemental legends.

Figures: Articles may have up to 10 main text figures. Figures must be prepared according to the policies outlined in our Instructions to Authors, under Data Presentation, https://jcb.rupress.org/site/misc/ifora.xhtml. All figures in accepted manuscripts will be screened prior to publication.

IMPORTANT: It is JCB policy that if requested, original data images must be made available. Failure to provide original images upon request will result in unavoidable delays in publication. Please ensure that you have access to all original microscopy and blot data images before submitting your revision.

Supplemental information: There are strict limits on the allowable amount of supplemental data. Articles may have up to 5 supplemental figures. Up to 10 supplemental videos or flash animations are allowed. A summary of all supplemental material should appear at the end of the Materials and methods section.

Please note that JCB now requires authors to submit Source Data used to generate figures containing gels and Western blots with all revised manuscripts. This Source Data consists of fully uncropped and unprocessed images for each gel/blot displayed in the main and supplemental figures. For assays performed using capillary electrophoresis and/or immunoassay-based detection, authors should instead provide the electropherogram graph(s) for each experiment, plotting fluorescence/chemiluminescence intensity vs. molecular weight/size. Please be sure to provide one Source Data file for each figure gels, blots, and/or capillary electrophoresis assays along with your revised manuscript files. File names for Source Data figures should be alphanumeric without any spaces or special characters (i.e., SourceDataF#, where F# refers to the associated main figure number or SourceDataFS# for those associated with Supplementary figures). For traditional gels and blots, the lanes of the gels/blots should be labeled as they are in the associated figure, the place where cropping was applied should be marked (with a box), and molecular weight/size standards should be labeled wherever possible. For capillary electrophoresis assays, each trace in the graph should be color-coded and labeled to indicate which protein, gene, or sample is being measured (please try to avoid red/green combinations to accommodate our color-blind readers).

Source Data files will be made available to reviewers during evaluation of revised manuscripts and, if your paper is eventually published in JCB, the files will be directly linked to specific figures in the published article.

Source Data Figures should be provided as individual PDF files (one file per figure). Authors should endeavor to retain a minimum resolution of 300 dpi or pixels per inch. Please review our instructions for export from Photoshop, Illustrator, and PowerPoint here: https://rupress.org/jcb/pages/submission-guidelines#revised

The typical timeframe for revisions is three to four months. If you anticipate any difficulties in meeting this aforementioned revision time limit, please contact us and we can work with you to find an appropriate time frame for resubmission. Please note that papers are generally considered through only one revision cycle, so any revised manuscript will likely be either accepted or rejected.

When submitting the revision, please include a cover letter addressing the reviewers' comments point by point. Please also highlight all changes in the text of the manuscript.

We hope that the comments below will prove constructive as your work progresses. We would be happy to discuss them further once you've had a chance to consider the points raised in this letter.

Thank you for this interesting contribution to Journal of Cell Biology. You can contact us at the journal office with any questions at cellbio@rockefeller.edu.

Sincerely,

Arshad Desai, PhD
Monitoring Editor
Journal of Cell Biology

Dan Simon, PhD
Scientific Editor
Journal of Cell Biology

Reviewer #1 (Comments to the Authors (Required)):

Epigenetic inheritance of the centromeres is critical for accurate chromosome segregation. Centromeres are defined by CENP-A-containing nucleosomes, which recruit further CENP-A and trigger the assembly of kinetochores. Drosophila lacks CCAN proteins except CENP-C, presenting a simplified model. Previous studies in Drosophila mitosis show interdependence of centromere localization among CID (CENP-A), CENP-C and CAL1, revealing a positive loop among these proteins that leads to the epigenetic maintenance of centromeres across mitotic cell cycles. This study investigated these dependencies in male meiosis by tracking their centromere localization at different meiotic stages after RNAi of each protein. The main conclusion of this study is that a simple positive loop model among these three proteins does not explain the epigenetic inheritance of centromeres in male meiosis. This is a novel finding which will potentially uncover interesting biology. Combined with the high quality of the data which are well analyzed and quantified, this study is worth publishing, provided that the specific issues stated below are satisfactorily addressed. Beyond that, it contains valuable observations, but they do not lead to an alternative model or hypothesis to explain the epigenetic inheritance of centromeres during Drosophila male meiosis.

The authors draws several conclusions based on differences observed between two different drivers, including the "two distinct pools" model. These observations are potentially interesting, but the conclusions seem to rely on the assumption that the different results using bam-GAL4 and Rbp4-GAL4 RNAi arise solely from difference in the timing of dsRNA expression (ie, whether these genes are silenced at the CC8 stage). However, this interpretation needs more caution. GAL4 drivers and RNAi should not be considered simply as on or off; there may be additional subtleties. Different drivers exhibit different GAL4 expression levels, and for each driver the GAL4 level may change at different meiotic stages even when it is "on". Therefore, the depletion levels of the target protein may also differ between the two drivers, rather than the differences are limited to the timing of RNAi onset. The differences in results from the two drivers may be influenced by differences in the residual amount of the target protein and by the sensitivity of loading efficiency to reduced protein levels.

Specific comments:
Figures: Figures should be clearly labelled with stages. It is frustrating for readers to have to refer to the legends each time.
Fig1C. It may be better to show CID intensity per DNA or haploid genome over the course of meiosis, rather than per nucleus, or show both.
L130 " with each providing higher than expected quantities (2.5-fold measured increases compared to the expected 2-fold)". The difference between 2 and 2.5 appears modest. This requires statistical analysis.
L154 "at the S1 stage, CAL1 is required for the stability of both nucleosomal and soluble CID". The rationale behind the conclusion about soluble CID is unclear, as only the CID intensity at centromeres was measured.
L160-166: "only 35% of the total CID population at centromeres is replenished in prophase I". If the CID intensity shows a 35% reduction, I think that (50-35)/50 =30% would be replenished, not 35%.
L178 "demonstrate that CENP-C localisation in meiosis is not strictly dependent on CID or CAL1." CAL1 is virtually lost from centromeres at S1 in CID or CAL1 RNAi, but substantial amounts of CID remained at S1 or prometaphase I. It is difficult to conclude whether CENP-C localisation is not dependent on CID.
L251: "The second post-meiotic phase of CID assembly cannot recover insufficiencies in the first phase." I could not fully follow the logic. In this set up, dsRNA was continuously expressed during both phases of CID assembly. Therefore I am not sure how it is possible to draw conclusions about the second phase of CID assembly, unless expression of these genes is induced at later stages.
L260: "cells are unable to transcribe new RNA". RNAseq only measures the amount of RNA, not the synthesis. RNA

degradation can regulate RNA levels even with continuous transcription.
L266 -268: "CID remains reduced" "both CID and CENP-C RNAi showed a further reduction in CID". Comparing % reductions from the control between two different stages is misleading, as there is a 100% increase in CID intensity (per DNA or haploid genome) due to new post-meiotic recruitment between these stages. For example, the CID intensity at centromeres (per DNA or haploid genome) was not reduced between these two stages but actually increased by 45%.
L268-274. "a further reduction in CID from the levels previously established in prometaphase I (Figure 2G), whereas CAL1 RNAi did not." This conclusion needs statistical analysis.
L345 "it (CenpC) is no longer required for CID assembly" I am not sure which data this conclusion is based on. RNAi cannot say that it is not required, especially when a significant amount (>50%) of CenpC remains at centromeres after RNAi.

Reviewer #2 (Comments to the Authors (Required)):

This study investigates the dynamics and interdependencies of core centromere proteins in Drosophila spermatogenesis, making several unique observations about centromere assembly. CID (Drosophila centromere histone) is assembled in two phases in meiosis. The authors find that the canonical assembly pathway, via the cognate histone chaperone CAL1 and the targeting unit CENP-C, is active in the first but not the second phase. In addition, CENP-C, generally localizes in a CID and CAL1 dependent manner , appears to be localizing independent of these factors in male meiosis. The second phase of assembly is independent of CAL1, an unexpected and surprising finding along with a few other new observations 1) CENP-C localization to centromere independent of CID levels, and 2) CID translation uncoupled from its deposition. These findings are curious, interesting, and potentially an exciting advance in the field, especially the possibility of a germline specific CID chaperone for assembly or chaperone independent pathway that is not known in other cell types. However, the use of RNAi lines and their efficacy, and somewhat inconsistent presentation of the data currently hinders interpretation and robust support of some conclusions. For e.g. to assess loading per se, we need to review fold-change in CID levels across stages - as shown in an early figure - consistently throughout the manuscript, which is not how it is presented now. There are also a few instances where the extent of the phenotype is hard to connect to the RNAi and its impacts. In addition, as noted below, some of these factors also impact stability and new assembly should be presented in that context altogether. These should be addressed in the manuscript. The findings are very interesting but require more clarification, support, and detail as it is a very new phenomenon.
Major comments:
1. The RNAi lines in Figure 2 are only compared to controls at a single stage (S1 or S6) but not across stages within each RNAi line. CID increases significantly, ~2.5 fold, from S1 - S6 (Fig 1C) in controls. To understand how loading is changed, S1 - S6 timepoints should be compared for the 3 RNAi lines, just like in Fig. 1C, and the fold-change compared to the control. This will visually address new loading in each line, especially as each RNAi line starts lower than controls at S1, which is the beginning of deposition. If assembly is impacted, the S1-S6 will not show a 2.5-fold difference in the RNAi. If there is no new phase I assembly in the absence of CAL1, then S1-S6 should show very low fold-change in the RNAi line. This data is already there in the paper and should be directly compared to each other (2A to 2F). This will be much easier to interpret and conserves the consistency of representing new loading while controlling for decreases due to stability issues (see point 3 later).
2. CENP-C localization despite a drop in CID is interesting and appears to be unique to the germline. The authors mention this could be due to dimerization or saturating binding sites of existing CID. Does CENP-C in Drosophila have binding domains for H3 as well? Is this true only for RNAi lines (stage specific knockdowns) or are there any hypomorphs of CID that show the same compensation of CENP-C localization?
3. In line 165 the authors note that 35% of CID is replenished at S6 representing underloading due to incomplete RNAi but CAL1 at least looks to be almost gone in CID and CAL1 RNAi (2D) so this is unlikely? Also, how would the authors place this modest 35% replenishment in the context of 2.5-fold total new assembly? Also, the RNAi lines start at ~30-35% lower than WT at S1 due to decreased stability which should be accounted for at S6? This part is confusing and can be explained better in the context of turnover of existing CID vs. assembly of new CID, and of course in case of the RNAi - assembly of new CID onto a reduced template due to decreased stability.
4. 3A, B is surprising. CENP-C is only reduced ~25% in prometaphase I. But Spc105 is nearly gone - seems to be ~15% from the graph. How do the authors reconcile this big difference? Is a large proportion of CENP-C at the centromeres not competent for binding the kinetochore? It is intriguing to consider that perhaps the bulk of CENP-C is required for second phase assembly, and/or compensation for any CID loss, while a subset of CENP-C is canonically bound to CID and required for kinetochore binding and decreases with CID proportionally just like other cell types. It would help if the percentage reduction of Spc105 was mentioned in the text (line 189) like Figure 2.
5. For Figure 5, T1-T5 should be compared per line to assess loading. It does seem strange that CENP-C levels are going down while CID is still assembling, especially as CAL1 seems to have no role in this stage. Is loading gradual in this window or happens all at once? That could explain why CENP-C is not needed once CID is loaded.
6. Overall, one major comment would be consistent presentation of data to enhance understanding, especially for the generalist reader. For e.g. as mentioned earlier, all loading experiments should be presented like 1F, which is impactful visually to demonstrate loading (only S1-S6 and T1-T5 is required in latter figures per RNAi line). Second, the text could be consistent in reporting percentage reductions as denoted in the graphs in the main text as followed in Fig. 2. This is useful as the reader does not have to refer to the graph axes to infer reductions each time. Third, reminding the generalist reader of the difference between decreases due to stability (turnover) vs. template-dependent new assembly and explaining the data in that context will elevate the manuscript.

Minor comments:
1. Is the high level of mis-segregation and no fertility phenotype surprising? CENP-C RNAi lines have quite high rates of errors. Is there a reduction in sperm count and the progeny are from the other intact sperm? Are there any other examples where high rates of mis segregation do not impact fertility?
2. The stages for each set of image panels should be on the figure (S1, T1, T5 etc.), specifically for figures where post-meiotic stages are ahead of prometaphase stages like Fig. 5. This is fine if they are labeled.
3. Line 220 appears unfinished, and Figure 5 legend needs (B) to be bolded.

Reviewer #3 (Comments to the Authors (Required)):

The manuscript by Keegan and Dunleavy describe the localization and potential regulation of CID (CENP-A) loading events that occur during sperm maturation in Drosophila melanogaster. CID is a conserved histone variant that is found at centromeric chromatin and is maintained on chromatin during the histone - protamine exchange during spermatogenesis. The loading machinery in Drosophila consist of the loading factor Cal1 and of the sole CCAN component of the inner kinetochore called CENP-C. Wereas the role of these factors in CID loading is well understood, their roles in meiotic cell division is less understood. Loading of CID accurs in prophase I and after meiosis II. CAL1 has not been detected in meiosis II, whereas CENP-C is detectable until meiosis II, leading to the authors' research question of how CID is loaded during or after meiosis II.
This manuscript advances our understanding of a role of CENP-C in CID loading during meiosis II when the loading factor CAL1 seems low or absent from centromeres. It starts with a brief introduction of CID loading and what is known about meiotic loading of CID and its orthologs in other species during meiosis. A brief section of what the manuscript shows maybe added to the end of the introduction.
The result section starts with a very nice graph of spermatogenesis in flies and what is known about the levels of CID, CENP-C and CAL1. However, in supplemental data S3E, the authors show that the transcription levels and dynamics of CENP-C and CAL1 are virtually identical. This would mean that CAL1 protein is either less stable than CENP-C or that it is activeley degraded to archive the differences in protein levels that the authors describe. None of this was tested but may be a very important aspect for this manuscript. As a side note, the authors may also show CID mRNA dynamics in supplemetal figure S3E (it is mentioned in the text).
Importantly, in Figure 1B, the authors measure the total anti-CID antibody fluorescence of the entire nucleus of a Z-projection with maximum intensity. The 16CC (S6) stands our as the by far most brightly stained. However, the nucleus is also at least 10 fold bigger (this is a guess from the size of the projected image) than any other stage of spermatogenesis. One could argue that the staining is brighter because of the size of the nucleus, background issues (antibodies are notoriously noisy, especially in the germ line), changes in chromatin structures etc. This needs to be excluded by for instance comparing it to a chromatin-associated staining that isn't suppose to change or other controlls that take these aspects into account.
This is especially important at the later stages when the authors observe the second waive of CID loading between stage 32CC, 64CC (T1) and 64CC (T5). 64CC (T1) nuclei are bigger (twice as big?), which fits with the measured increase in signal intensity and may be due to changes in nuclear size or chromatin compaction rather than more CID loading. If the authors want to measure CID loading, a SNAP pulse chase experiments is required as has been done before with human CENP-A loading or other inducible systems that work well in flies (Gal4-UAS system as used in later experiments for inducing RNAi). The experiments provided in this manuscript cannot distinguish loading and pre-exsisting proteins. A simple solution here is probably more careful wording.
In Figure 2 the authors use Gal4-UAS to induce RNA interference specifcally in the germ-line specific bam-Gal4 driver. CID was significantly reduced in CAL1 RNAi at stage S1, which the authors interpret that CAL1 is required for the stability of already loaded CID since CID RNAi at this stage does not affect the levels of CID at this stage. Alternativeley, CAL1 could be involved in loading of pre-existing CID proteins. Again to disriminate this, the authors would need an inducible system. From the presented data, it is unclear if RNAi of CID and CENP-C is actually functional.The authors claim that this is because there is no loading, which could be the case of course but it could also be that the RNAi doesn't work or is insufficiently effective at this stage or with the used RNAi fly lines in combination with the used Gal4 drivers (The authors point out that they do not know how efficient the RNAi knowdowns were (line 167). Measuring the levels of mRNA by qPCR would be a way around this problem and strengthening their claims (even if this does not mean that the porteins levels are reduced either). Generally, knockdown efficiencies need to be measured throughout the paper. The authors also claim that CAL1 localizes to the nucleolus in Figure 2D and 2E. No nucleolar marker was used to substantiate this claim.
In Figure 3, the authors show that the outer kinetochore component SPC105 is affected by CENP-C RNAi but not by CID or CAL1. Again, CID signal wasn't affected by CID RNAi (perhaps due to no loading at this stage but this isnt shown) so the most obvious conclusion is that there is sufficient CID protein left to recuit SPC105. The authors then nicely show that CENP-C is required for SPC105 recuitment and show nice rescue experiments. Also for the bipolar spidle assay, CENP-C is clearly required, which is a nice results. However, as long as they don't show that the levels of CID and CAL1 significantly change, they cannot claim that these proteins arent required for bipolar spindle alignment only that CENP-C IS required. In fact, even reduced levels of these proteins may be sufficient to form a bipolar spindle. The last sentence in this paragraph (line 211) is unsubstantiated: "Additionally , a definciency in CID and CAL1 can be tolerated,...." The authors did not do double known downs of CAL1 and CID, the 'and' should be 'or' unless they do double knockdowns.
From Figure 4A, it appears as if the AATAC repeat is on the Y chromosome, the 359 bp repeat on the X chromsome, the text (line 218) states it the other way around, please clarify what is correct. These data are very nice but again, if CAL1 and/or CID

KD was insufficient to affect protein levels, the claims that CENP-C is the most important factor of the three (CID, CAL1, CENP-C) may be based on a more efficient RNAi for CENP-C.

In Figure 5, the authors show that when CID assembly is disrupted in the first phase, the second assembly phase is insufficient to rescue the assembly. Here, the authors also need to show if CAL1 RNAi with Rbp4 Gal4 (and the other RNAi experiments) are causing an efficient knowckdown or wether the differences in the requirenment of CAL1 is due to an insufficient depletion by Rbp4 Gal4. The lack of any fertility defects could also be an indication that the residual proteins after knockdown is sufficient to allow sperm maturation.

Overall, the paper reads nicley and the figures are well presented and the work is very interesting. If the authors can provide data on the RNAi efficiencies for the different proteins and drivers and find ways to distinguish loading from pre-excisting proteins (or tone it down), this will be a very nice study. The reviewer is aware that this requires new experiments. Many may be done by qPRC and better controlled quantifications of the immunofluorescence images as outlined above. However, their claims need more quantitative and better controlled experiments.

Rebuttal for Keegan et al.

Reviewer #1 comments (shown in blue; responses in black)

The main conclusion of this study is that a simple positive loop model among these three proteins does not explain the epigenetic inheritance of centromeres in male meiosis. This is a novel finding which will potentially uncover interesting biology. Combined with the high quality of the data which are well analyzed and quantified, this study is worth publishing, provided that the specific issues stated below are satisfactorily addressed. Beyond that, it contains valuable observations, but they do not lead to an alternative model or hypothesis to explain the epigenetic inheritance of centromeres during Drosophila male meiosis.

To highlight the overall significance of our findings, we have added the following to the Discussion on p8: *we confirm and refine the requirements for two unique centromere assembly events in meiosis that ultimately ensure paternal centromeres harbour sufficient CID for embryogenesis.*

The authors draws several conclusions based on differences observed between two different drivers, including the "two distinct pools" model. These observations are potentially interesting, but the conclusions seem to rely on the assumption that the different results using bam-GAL4 and Rbp4-GAL4 RNAi arise solely from difference in the timing of dsRNA expression (ie, whether these genes are silenced at the CC8 stage). However, this interpretation needs more caution. GAL4 drivers and RNAi should not be considered simply as on or off; there may be additional subtleties. Different drivers exhibit different GAL4 expression levels, and for each driver the GAL4 level may change at different meiotic stages even when it is "on". Therefore, the depletion levels of the target protein may also differ between the two drivers, rather than the differences are limited to the timing of RNAi onset. The differences in results from the two drivers may be influenced by differences in the residual amount of the target protein and by the sensitivity of loading efficiency to reduced protein levels.

We thank the reviewer for raising this important point. To address this possibility, we have added the following sentence to p7: *Although, in addition to timing differences, we cannot exclude that the amount of GAL4 expression differs between the drivers.*

Specific comments:

Figures: Figures should be clearly labelled with stages. It is frustrating for readers to have to refer to the legends each time.

We have re-labelled all figures indicating the stage.

Fig1C. It may be better to show CID intensity per DNA or haploid genome over the course of meiosis, rather than per nucleus, or show both.

For improved clarity, we now indicate the ploidy (2n or n) and DNA content for specific cell stages on Figure 1C.

L130 " with each providing higher than expected quantities (2.5-fold measured increases compared to the expected 2-fold)". The difference between 2 and 2.5 appears modest. This requires statistical analysis.

We have carried out statistics on this data which is significantly different, p3: *This result indicates that despite two rounds of chromosome segregation in meiosis, CID level in haploid spermatids is comparable to diploid pre-meiotic cells due to two unique phases of CID assembly occurring during spermatogenesis, with the first providing significantly higher than expected quantities (2.5-fold measured increases compared to the expected 2-fold).*

L154 "at the S1 stage, CAL1 is required for the stability of both nucleosomal and soluble CID". The rationale behind the conclusion about soluble CID is unclear, as only the CID intensity at centromeres was measured.

We agree with this point. We have modified this statement as follows on p4: *In summary, at the S1 stage, CAL1 is required for the stability of nucleosomal CID, and CENP-C can localise to centromeres in a manner that is not strictly dependent on CID and CAL1 level.*

L160-166: "only 35% of the total CID population at centromeres is replenished in prophase I". If the CID intensity shows a 35% reduction, I think that (50-35)/50 =30% would be replenished, not 35%.
For improved clarity, we have now expressed these values as 'fold changes', and have included a new data panel on this comparing CID level at S1, S6, T1 and T5 stages in the control and RNAi lines (Figure S2C). We refer to this data on p4: *A 2.5-fold increase in total CID normally occurs in prophase I (Figure 1C). CID, CAL1, and CENP-C RNAi display 1.7, 2.1, and 1.7-fold changes in CID, respectively, indicating that new CID assembly is affected in all three lines (Figure S2C).*
In addition, we now express the second loading event as a fold change, p3: *Consistent with previous findings of a second phase of CID assembly in post-meiotic spermatids (Dunleavy et al., 2012), an increase in CID intensity (1.8-fold) was observed between T1 and T5 stages.*

L178 "demonstrate that CENP-C localisation in meiosis is not strictly dependent on CID or CAL1." CAL1 is virtually lost from centromeres at S1 in CID or CAL1 RNAi, but substantial amounts of CID remained at S1 or prometaphase I. It is difficult to conclude whether CENP-C localisation is not dependent on CID.
We agree with this point. We have modified this statement as follows on p4/p5: *Taken together, analyses of both early (S1) and late (prometaphase I) stages demonstrate that CENP-C localisation in meiosis is not strictly dependent on CAL1, and can occur normally even with reduced CID.*

L251: "The second post-meiotic phase of CID assembly cannot recover insufficiencies in the first phase." I could not fully follow the logic. In this set up, dsRNA was continuously expressed during both phases of CID assembly. Therefore I am not sure how it is possible to draw conclusions about the second phase of CID assembly, unless expression of these genes is induced at later stages.
We have modified the title of the results section on p6 as follows: *The second post-meiotic phase of CID assembly cannot recover <u>deficient assembly</u> in the first phase.*
As stated on page 4, the *bam-GAL4* driver is active during the first phase of assembly. Our results show that the second phase of assembly (in 64CC) is also impacted despite the shutdown of bam-GAL4 expression in 16CCs.
To improve clarity, we now refer to bam-GAL4 expression timing again on p6: *The bam-GAL4 driver is expressed in 8CC and 16CC until late prophase I (S6).*

L260: "cells are unable to transcribe new RNA". RNAseq only measures the amount of RNA, not the synthesis. RNA degradation can regulate RNA levels even with continuous transcription.
This is true. We have removed this line.

L266 -268: "CID remains reduced" "both CID and CENP-C RNAi showed a further reduction in CID". Comparing % reductions from the control between two different stages is misleading, as there is a 100% increase in CID intensity (per DNA or haploid genome) due to new post-meiotic recruitment between these stages. For example, the CID intensity at centromeres (per DNA or haploid genome) was not reduced between these two stages but actually increased by 45%.
We apologise for any confusion here. For each of these statements, we are referring to CID levels relative to control at each stage and are not directly comparing levels across stages.

L268-274. "a further reduction in CID from the levels previously established in prometaphase I (Figure 2G), whereas CAL1 RNAi did not." This conclusion needs statistical analysis.
We have completed statistical analysis on this data to confirm significance, p6: *Interestingly, both CID and CENP-C RNAi showed a further significant reduction in CID from the levels previously established in prometaphase I (Figure 2G), whereas CAL1 RNAi did not.*

L345 "it (CenpC) is no longer required for CID assembly" I am not sure which data this conclusion is based on. RNAi cannot say that it is not required, especially when a significant amount (>50%) of CenpC remains at centromeres after RNAi.

This statement is based on the finding that Rbp4-GAL4 driver experiments show a depletion of CENP-C with no impact on CID (shown in Figure 5D and 5G). We have now toned down this point in the Discussion on p8: *At this point, CENP-C continues to be recruited to the centromere, but it is not critical for complete CID assembly*.

Reviewer #2 comments (shown in blue; responses in black)

These findings are curious, interesting, and potentially an exciting advance in the field, especially the possibility of a germline specific CID chaperone for assembly or chaperone independent pathway that is not known in other cell types. However, the use of RNAi lines and their efficacy, and somewhat inconsistent presentation of the data currently hinders interpretation and robust support of some conclusions. For e.g. to assess loading per se, we need to review fold-change in CID levels across stages - as shown in an early figure -consistently throughout the manuscript, which is not how it is presented now. There are also a few instances where the extent of the phenotype is hard to connect to the RNAi and its impacts. In addition, as noted below, some of these factors also impact stability and new assembly should be presented in that context altogether. These should be addressed in the manuscript. The findings are very interesting but require more clarification, support, and detail as it is a very new phenomenon.

Major comments:

1. The RNAi lines in Figure 2 are only compared to controls at a single stage (S1 or S6) but not across stages within each RNAi line. CID increases significantly, ~2.5 fold, from S1 - S6 (Fig 1C)in controls. To understand how loading is changed, S1 - S6 timepoints should be compared for the 3 RNAi lines, just like in Fig. 1C, and the fold-change compared to the control. This will visually address new loading in each line, especially as each RNAi line starts lower than controls at S1, which is the beginning of deposition. If assembly is impacted, the S1-S6 will not show a 2.5-fold difference in the RNAi. If there is no new phase I assembly in the absence of CAL1, then S1-S6 should show very low fold-change in the RNAi line. This data is already there in the paper and should be directly compared to each other (2A to 2F). This will be much easier to interpret and conserves the consistency of representing new loading while controlling for decreases due to stability issues (see point 3 later).

We now include an entirely new data set and quantitation showing fold changes in the control and RNAi lines at both loading phases (Figure S2C). We also provide qPCR data to confirm RNAi knockdown efficiencies using the two drivers (Figure S2A, S2B).

2. CENP-C localization despite a drop in CID is interesting and appears to be unique to the germline. The authors mention this could be due to dimerization or saturating binding sites of existing CID. Does CENP-C in Drosophila have binding domains for H3 as well? Is this true only for RNAi lines (stage specific knockdowns) or are there any hypomorphs of CID that show the same compensation of CENP-C localization?

There are no known H3 binding sites on Drosophila CENP-C. We have focused our study on stage-specific RNAi depletions, and have not examined CENP-C localisation in *cid* mutants. Given that CID is essential for mitosis, this might complicate analyses in the germline.

3. In line 165 the authors note that 35% of CID is replenished at S6 representing underloading due to incomplete RNAi but CAL1 at least looks to be almost gone in CID and CAL1 RNAi (2D) so this is unlikely? Also, how would the authors place this modest 35% replenishment in the context of 2.5-fold total new assembly? Also, the RNAi lines start at ~30-35% lower than WT at S1 due to decreased stability which should be accounted for at S6? This part is confusing and can be explained better in the context of turnover of existing CID vs. assembly of new CID, and of course in case of the RNAi - assembly of new CID onto a reduced template due to decreased stability.

We have removed statements of such percentages. To improve clarity and interpretation of effects on loading, we now include an entirely new data set showing fold change in CID in control and RNAi lines (Figure S2C).

4. 3A, B is surprising. CENP-C is only reduced ~25% in prometaphase I. But Spc105 is nearly gone - seems to be ~15% from the graph. How do the authors reconcile this big difference? Is a large proportion of CENP-C at the centromeres not competent for binding the kinetochore? It is intriguing to consider that perhaps the bulk of CENP-C is required for second phase assembly, and/or compensation for any CID loss, while a subset of CENP-C is canonically bound to CID and required for kinetochore binding and decreases with CID proportionally just like other cell types. It would help if the percentage reduction of Spc105 was mentioned in the text (line 189) like Figure 2.
We agree with this point. To highlight this, we have indicated the median percentage of Spc105 that remains upon CENP-C RNAi in Figure 3B and legend.

5. For Figure 5, T1-T5 should be compared per line to assess loading. It does seem strange that CENP-C levels are going down while CID is still assembling, especially as CAL1 seems to have no role in this stage. Is loading gradual in this window or happens all at once? That could explain why CENP-C is not needed once CID is loaded.
T1-T5 loading is now compared for all lines based on the new Figure S2C. Based on our previous publication, this loading appears to be gradual and correlates with the gradual disappearance of CENP-C (Dunleavy et al. 2012, PLoS Genetics).

Overall, one major comment would be consistent presentation of data to enhance understanding, especially for the generalist reader. For e.g. as mentioned earlier, all loading experiments should be presented like 1F, which is impactful visually to demonstrate loading (only S1-S6 and T1-T5 is required in latter figures per RNAi line).
To supplement Figure 1C, we have added the quantitation of an entirely new data set (Figure S2C) that allows us to compare the fold change in CID across all stages and RNAi knockdowns.

Second, the text could be consistent in reporting percentage reductions as denoted in the graphs in the main text as followed in Fig. 2. This is useful as the reader does not have to refer to the graph axes to infer reductions each time.
We have added all percentages directly to each graph.

Third, reminding the generalist reader of the difference between decreases due to stability (turnover) vs. template-dependent new assembly and explaining the data in that context will elevate the manuscript
We have carefully revised the language used throughout the manuscript to ensure that effects on stability, assembly and maintenance are clear.

Minor comments:
Is the high level of mis-segregation and no fertility phenotype surprising? CENP-C RNAi lines have quite high rates of errors. Is there a reduction in sperm count and the progeny are from the other intact sperm? Are there any other examples where high rates of mis segregation do not impact fertility?
This is indeed a surprising result. Although, Drosophila spermiogenesis mutant phenotypes are quite variable in terms of fertility and sterility effects (see recent review from Dubruille et al. 2025, Current Opinions in Genetics and Development). We suppose that CENP-C RNAi lines are fertile as they do manage to produce normal sperm. We now include a quantitation indicating the presence of mature sperm in the seminal vesicles (Figure 4F, see legend).

2. The stages for each set of image panels should be on the figure (S1, T1, T5 etc.), specifically for figures where post-meiotic stages are ahead of prometaphase stages like Fig. 5. This is fine if they are labeled.

All stages are now clearly labelled on each figure.

3. Line 220 appears unfinished, and Figure 5 legend needs (B) to be bolded.
This is now fixed.

Reviewer #3 comments (shown in blue; responses in black)

A brief section of what the manuscript shows maybe added to the end of the introduction.
We believe this information is effectively highlighted in the abstract: *In early prophase I, CENP-C functions with CAL1 to maintain the centromere. In late prophase I, CAL1 is undetectable at centromeres and CENP-C is not critical for centromere maintenance. Instead, CENP-C is crucial for meiotic kinetochore recruitment and function. This CENP-C pool also functions in CID assembly post-meiosis II, which is independent of CAL1.*

The result section starts with a very nice graph of spermatogenesis in flies and what is known about the levels of CID, CENP-C and CAL1. However, in supplemental data S3E, the authors show that the transcription levels and dynamics of CENP-C and CAL1 are virtually identical. This would mean that CAL1 protein is either less stable than CENP-C or that it is activeley degraded to archive the differences in protein levels that the authors describe. None of this was tested but may be a very important aspect for this manuscript. As a side note, the authors may also show CID mRNA dynamics in supplemetal figure S3E (it is mentioned in the text).
Previous studies in mitosis have shown that while CID and CENP-C levels decrease at 50% each cell cycle, CAL1 displays high turnover (Mellone et al. 2011, PLoS Genetics). CID mRNA dynamics were not included in Figure S4E as there were insufficient data available from the Fly Cell Atlas for each stage .

Importantly, in Figure 1B, the authors measure the total anti-CID antibody fluorescence of the entire nucleus of a Z-projection with maximum intensity. The 16CC (S6) stands our as the by far most brightly stained. However, the nucleus is also at least 10 fold bigger (this is a guess from the size of the projected image) than any other stage of spermatogenesis. One could argue that the staining is brighter because of the size of the nucleus, background issues (antibodies are notoriously noisy, especially in the germ line), changes in chromatin structures etc. This needs to be excluded by for instance comparing it to a chromatin-associated staining that isn't suppose to change or other controlls that take these aspects into account. This is especially important at the later stages when the authors observe the second waive of CID loading between stage 32CC, 64CC (T1) and 64CC (T5). 64CC (T1) nuclei are bigger (twice as big?), which fits with the measured increase in signal intensity and may be due to changes in nuclear size or chromatin compaction rather than more CID loading.
The possibility that changes in CID intensity are related to changes in chromatin structure and antibody accessibility was addressed in previous publications (Dunleavy et al. 2012 and Raychaudhuri et al. 2012). Both studies showed comparable changes in CID intensity, reflective of CID assembly, could be observed using anti-CID antibody staining and direct imaging of GFP-tagged CID.

If the authors want to measure CID loading, a SNAP pulse chase experiments is required as has been done before with human CENP-A loading or other inducible systems that work well in flies (Gal4-UAS system as used in later experiments for inducing RNAi). The experiments provided in this manuscript cannot distinguish loading and pre-exsisting proteins. A simple solution here is probably more careful wording. In Figure 2 the authors use Gal4-UAS to induce RNA interference specifcally in the germ-line specific bam-Gal4 driver. CID was significantly reduced in CAL1 RNAi at stage S1, which the authors interpret that CAL1 is required for the stability of already loaded CID since CID RNAi at this stage does not affect the levels of CID at this stage. Alternativeley, CAL1 could be involved in loading of pre-existing CID proteins. Again to disriminate this, the authors would need an inducible system. From the presented data, it is unclear if RNAi of CID and CENP-C is actually functional. The authors claim that this is because there is no loading, which could be the case of course but it could also be that the RNAi doesn't work or is insufficiently effective at this stage or with the used RNAi fly lines in combination

with the used Gal4 drivers (The authors point out that they do not know how efficient the RNAi knowdowns were (line 167). Measuring the levels of mRNA by qPCR would be a way around this problem and strengthening their claims (even if this does not mean that the porteins levels are reduced either). Generally, knockdown efficiencies need to be measured throughout the paper.

We agree that SNAP pulse chase experiments that enable labelling of the newly synthesized CID pool would be powerful. Indeed, SNAP-tagged CID lines were developed in the lab precisely for this purpose. However, the efficiency of TMR labelling and wash out was not very effective in tissues, and therefore this approach was deemed unsuitable. As an alternative approach, we have included a new data set that allows us to compare the fold change in CID intensity at each stage in the control and RNAi lines (Figure S2C). Here, we interpret fold changes in CID intensity as CID assembly, which can be directly compared for both loading phases. To address the efficiency of knockdowns, we have now added additional qPCR data confirming respective depletions in bam-GAL4 and Rbp4-GAL4 experiments (Figure S2A, S2B).

The authors also claim that CAL1 localizes to the nucleolus in Figure 2D and 2E. No nucleolar marker was used to substantiate this claim.

We now cite Kwenda et al. 2016 which showed CAL1 localisation to centromeres and nucleoli in Drosophila spermatocytes.

In Figure 3, the authors show that the outer kinetochore component SPC105 is affected by CENP-C RNAi but not by CID or CAL1. Again, CID signal wasn't affected by CID RNAi (perhaps due to no loading at this stage but this isnt shown) so the most obvious conclusion is that there is sufficient CID protein left to recuit SPC105. The authors then nicely show that CENP-C is required for SPC105 recuitment and show nice rescue experiments. Also for the bipolar spidle assay, CENP-C is clearly required, which is a nice results. However, as long as they don't show that the levels of CID and CAL1 significantly change, they cannot claim that these proteins aren't required for bipolar spindle alignment only that CENP-C IS required. In fact, even reduced levels of these proteins may be sufficient to form a bipolar spindle. The last sentence in this paragraph(line 211) is unsubstantiated: "Additionally , a defninciency in CID and CAL1 can be tolerated,...."The authors did not do double known downs of CAL1 and CID, the 'and' should be 'or' unless they do double knockdowns.

We apologise for any confusion. In Figure 2G, we show that at prometaphase I, CID is affected in both CID and CAL1 RNAi. We hope that by adding labels indicating specific stages to all images and by indicating percentages directly on graphs, these findings are more clear.

On page 5, we have changed this to 'or': *Additionally, a deficiency in CID or CAL1 can be tolerated, providing that sufficient CENP-C is recruited.*

From Figure 4A, it appears as if the AATAC repeat is on the Y chromosome, the 359 bp repeat on the X chromsome, the text (line 218) states it the other way around, please clarify what is correct.

Thanks. We have fixed this.

These data are very nice but again, if CAL1 and/or CID KD was insufficient to affect protein levels, the claims that CENP-C is the most important factor of the three (CID, CAL1,CENP-C) may be based on a more efficient RNAi for CENP-C. In Figure 5, the authors show that when CID assembly is disrupted in the first phase, the second assembly phase is insufficient to rescue the assembly. Here, the authors also need to show if CAL1 RNAi with Rbp4 Gal4 (and the other RNAi experiments) are causing an efficient knowckdown or wether the differences in the requirenment of CAL1 is due to an insufficient depletion by Rbp4 Gal4.

We now provide qPCR data to confirm RNAi knockdown efficiencies using the two drivers (Figure S2A, S2B).

The lack of any fertility defects could also be an indication that the is residual proteins after knockdown is sufficient to allow sperm maturation. Overall, the paper reads nicley and the figures are well presented and the work is very interesting. If the authors can provide data on the RNAi efficiencies for

the different proteins and drivers and find ways to distinguish loading from pre-excisting proteins (or tone it down),this will be a very nice study. The reviewer is aware that this requires new experiments. Many may be done by qPRC and better controlled quantifications of the immunofluorescence images as outlined above. However, their claims need more quantitative and better controlled experiments.
We believe we have addressed these points by including additional qPCR data confirming respective knockdowns and toning down the language. We also believe that the inclusion of a new Figure S2C that expresses changes in CID intensity as fold changes helps with the interpretation of data related to CID assembly.

May 26, 2026

RE: JCB Manuscript #202512053R

Elaine Dunleavy
Ollscoil na Gaillimhe - University of Galway

Dear Dr. Dunleavy,

Thank you for submitting your revised manuscript entitled "Distinct pools of CENP-A and CENP-C support unique phases of centromere assembly in spermatogenesis." The manuscript was re-assessed by two of the original reviewers. We would be happy to publish your paper in JCB pending final revisions necessary to meet our formatting guidelines (see details below).

Please consider Reviewer #1's final comment and adjust the title. We also agree with Reviewer #2 that the graph in figure S2C should be moved to a main figure.

To avoid unnecessary delays in the acceptance and publication of your paper, please read the following information carefully.

A. MANUSCRIPT ORGANIZATION AND FORMATTING:

Full guidelines are available on our Instructions for Authors page, https://jcb.rupress.org/submission-guidelines#revised.
Submission of a paper that does not conform to JCB guidelines will delay the acceptance of your manuscript.

1) Text limits: Character count for Articles is < 40,000, not including spaces. Count includes title page, abstract, introduction, results, discussion, and acknowledgments. Count does not include materials and methods, figure legends, references, tables, or supplemental legends.

2) Figure formatting: Articles may have up to 10 main text figures. Scale bars must be present on all microscopy images, including inset magnifications. Also, please avoid pairing red and green for images and graphs to ensure legibility for color-blind readers. If red and green are paired for images, please ensure that the particular red and green hues used in micrographs are distinctive with any of the colorblind types. If not, please modify colors accordingly or provide separate images of the individual channels.

3) Statistical analysis: Error bars on graphic representations of numerical data must be clearly described in the figure legend. The number of independent data points (n) represented in a graph must be indicated in the legend. Please indicate whether 'n' refers to technical or biological replicates (i.e. number of analyzed cells, samples or animals, number of independent experiments). If independent experiments with multiple biological replicates have been performed, we recommend using distribution-reproducibility SuperPlots (please see Lord et al., JCB 2020) to better display the distribution of the entire dataset, and report statistics (such as means, error bars, and P values) that address the reproducibility of the findings.

Statistical methods should be explained in full in the materials and methods. For figures presenting pooled data the statistical measure should be defined in the figure legends. Please also be sure to indicate the statistical tests used in each of your experiments (both in the figure legend itself and in a separate methods section) as well as the parameters of the test (for example, if you ran a t-test, please indicate if it was one- or two-sided, etc.). Also, if you used parametric tests, please indicate if the data distribution was tested for normality (and if so, how). If not, you must state something to the effect that "Data distribution was assumed to be normal but this was not formally tested."

4) Title: The title should be less than 100 characters including spaces. Make the title concise but accessible to a general readership.

5) Materials and methods: Should be comprehensive and not simply reference a previous publication for details on how an experiment was performed. Please provide full descriptions (at least in brief) in the text for readers who may not have access to referenced manuscripts. The text should not refer to methods "...as previously described."

6) For all cell lines, vectors, strains, constructs/cDNAs, etc. - all genetic material: please include database / vendor ID (e.g. Addgene, ATCC, etc.) or if unavailable, please briefly describe their basic genetic features, even if described in other published work or gifted to you by other investigators (and provide references where appropriate). Please be sure to provide the sequences for all of your oligos: primers, si/shRNA, RNAi, gRNAs, etc. in the materials and methods. You must also indicate in the methods the source, species, and catalog numbers/vendor identifiers (where appropriate) for all of your antibodies, including secondary. If antibodies are not commercial, please add a reference citation if possible.

7) Microscope image acquisition: The following information must be provided about the acquisition and processing of images:

a. Make and model of microscope
b. Type, magnification, and numerical aperture of the objective lenses
c. Temperature
d. Imaging medium
e. Fluorochromes
f. Camera make and model
g. Acquisition software
h. Any software used for image processing subsequent to data acquisition. Please include details and types of operations involved (e.g., type of deconvolution, 3D reconstitutions, surface or volume rendering, gamma adjustments, etc.).

8) References: There is no limit to the number of references cited in a manuscript. References should be cited parenthetically in the text by author and year of publication. Abbreviate the names of journals according to PubMed.

9) Supplemental materials: Articles may have up to 5 supplemental figures and 10 videos. Please also note that tables, like figures, should be provided as individual, editable files. A summary of all supplemental material should appear at the end of the Materials and methods section. Please include one brief sentence per item.

10) eTOC summary: A ~40-50 word summary that describes the context and significance of the findings for a general readership should be included on the title page. The statement should be written in the present tense and refer to the work in the third person. It should begin with "First author name(s) et al..." to match our preferred style.

11) Conflict of interest statement: JCB requires inclusion of a statement in the acknowledgements regarding competing financial interests. If no competing financial interests exist, please include the following statement: "The authors declare no competing financial interests." If competing interests are declared, please follow your statement of these competing interests with the following statement: "The authors declare no further competing financial interests."

12) A separate author contribution section is required following the Acknowledgments in all research manuscripts. All authors should be mentioned and designated by their first and middle initials and full surnames. We encourage use of the CRediT nomenclature (https://casrai.org/credit/).

13) ORCID IDs: ORCID IDs are unique identifiers allowing researchers to create a record of their various scholarly contributions in a single place. Please note that ORCID IDs are required for all authors. At resubmission of your final files, please be sure to provide your ORCID ID and those of all co-authors.

14) Journal of Cell Biology now requires a data availability statement for all research article submissions. These statements will be published in the article directly above the Acknowledgments. The statement should address all data underlying the research presented in the manuscript. Please visit the JCB instructions for authors for guidelines and examples of statements at (https://rupress.org/jcb/pages/editorial-policies#data-availability-statement).

B. FINAL FILES:

Please upload the following materials to our online submission system. These items are required prior to acceptance. If you have any questions, contact JCB's Managing Editor, Lindsey Hollander (lhollander@rockefeller.edu).

-- An editable version of the final text (.DOC or .DOCX) is needed for copyediting (no PDFs).

-- High-resolution figure and MP4 video files: See our detailed guidelines for preparing your production-ready images, https://jcb.rupress.org/fig-vid-guidelines.

-- Cover images: If you have any striking images related to this story, we would be happy to consider them for inclusion on the journal cover. Submitted images may also be chosen for highlighting on the journal table of contents or JCB homepage carousel. Images should be uploaded as TIFF or EPS files and must be at least 300 dpi resolution.

It is JCB policy that if requested, original data images must be made available to the editors. Failure to provide original images upon request will result in unavoidable delays in publication. Please ensure that you have access to all original data images prior to final submission.

The license to publish form must be signed before your manuscript can be sent to production. A link to the license to publish form will be sent to the corresponding author only. Please take a moment to check your funder requirements before choosing the appropriate license.

Additionally, JCB encourages authors to submit a short video summary of their work. These videos are intended to convey the main messages of the study to a non-specialist, scientific audience. Think of them as an extended version of your abstract, or a short poster presentation. We encourage first authors to present the results to increase their visibility. The videos will be shared

on social media to promote your work. For more detailed guidelines and tips on preparing your video, please visit https://rupress.org/jcb/pages/submission-guidelines#videoSummaries.

Thank you for your attention to these final processing requirements. Please revise and format the manuscript and upload materials within 14 days. If you need an extension for whatever reason, please let us know and we can work with you to determine a suitable revision period.

Please contact the journal office with any questions at cellbio@rockefeller.edu.

Thank you for this interesting contribution, we look forward to publishing your paper in Journal of Cell Biology.

Sincerely,

Arshad Desai, PhD
Monitoring Editor
Journal of Cell Biology

Dan Simon, PhD
Scientific Editor
Journal of Cell Biology

Reviewer #1 (Comments to the Authors (Required)):

The authors appropriately responded to my comments mostly by additional statistical analysis and softening the tone of the their claims. Although they have added a reservation statement for their two-pool model, their title or abstract has not been changed to reflect this. The two-pool model is a good working hypothesis that came out from this study, but I feel that this needs more evidence before considering a solid conclusion which can be used as the title. I think that it is fine to leave it in the abstract, but may not be suitable as the title of the paper.

Reviewer #2 (Comments to the Authors (Required)):

The most important part of the revision is Figure S2C. This figure clarifies some of the concerns regarding loading vs. stability (turnover of pre-existing proteins). I believe S2C nicely shows possible loading defects albeit tempered by the fact that some of it can be attributed to ongoing stability issues due to RNAi of key stability factors, but is convincing nonetheless. As noted by another reviewer, SNAP pulse chase experiments are the gold standard for nascent CENP-A assembly, however, given that this has already been tried (commendable effort) and may not work well in the germline, the current study is the next best approach. All other concerns have been met, especially as some of the questions which remain are perhaps beyond the scope of this immediate phenomenological discovery. One strong suggestion would be to move Figure S2C to a main figure as it bears the crux of distinguishing loading vs. stability and is very well done. Overall, this is a nice study and will be a great contribution to the field of centromere assembly, especially as these are usually hard to pursue in vivo. it also highlights the possibility of non-canonical assembly phases, which is a new concept.
